# Estimation of Lens Stray Light with Regard to the Incapacitation of Imaging Sensors

**DOI:** 10.3390/s20216308

**Published:** 2020-11-05

**Authors:** Gunnar Ritt, Bastian Schwarz, Bernd Eberle

**Affiliations:** Fraunhofer IOSB, Gutleuthausstr. 1, 76275 Ettlingen, Germany; bastian.schwarz@iosb.fraunhofer.de (B.S.); bernd.eberle@iosb.fraunhofer.de (B.E.)

**Keywords:** stray light, imaging sensor, scatter parameters, Harvey scatter model, laser safety

## Abstract

We present our efforts on estimating light scattering characteristics from commercial off-the-shelf (COTS) camera lenses in order to deduce thereof a set of generic scattering parameters valid for a specific lens class (double Gauss lenses). In previous investigations, we developed a simplified theoretical light scattering model to estimate the irradiance distribution in the focal plane of a camera lens. This theoretical model is based on a 3-parameter bidirectional scattering distribution function (BSDF), which describes light scattering from rough surfaces of the optical elements. Ordinarily, the three scatter parameters of the BSDF are not known for COTS camera lenses, which makes it necessary to assess them by own experiments. Besides the experimental setup and the measurement process, we present in detail the subsequent data exploitation. From measurements on seven COTS camera lenses, we deduced a generic set of scatter parameters. For a deeper analysis, the results of our measurements have also been compared with the output of an optical engineering software. Together with our theoretical model, now stray light calculations can be accomplished even then, when specific scatter parameters are not available from elsewhere. In addition, the light scattering analyses also allow considering the glare vulnerability of optical systems in terms of laser safety.

## 1. Introduction

Right after laser emission had been demonstrated the first time, the specific hazards of this new kind of light source became obvious [1,2]. Besides electrical risks associated with high voltage driven components, the main risk to individuals normally occurs through direct exposure of the eye to the laser beam. The worldwide efforts by a vast number of researchers to establish rules for the safe use of lasers led to the well-known laser safety standards like IEC 60825-1 or ANSI Z136.1 [3,4]. These standards provide quantities like *maximum permissible exposure* (MPE) limits, representing the highest level of irradiance or radiant exposure in order to enable a safe view into the laser beam for the human eye. The MPE depends on various parameters, like laser output power, wavelength, exposure time to the eye or pulse width and pulse repetition rate. Based on the MPE value and the beam divergence, the minimum distance between eye and laser source can be calculated, the so-called *nominal ocular hazard distance* (NOHD), below which the direct view into the laser beam is not safe. Recently, Williamson and McLin transferred the damage related MPE/NOHD concept to laser eye dazzle [5]. Equivalently to MPE and NOHD, they established the terms *maximum dazzle exposure* (MDE) and *nominal ocular dazzle distance* (NODD). Due to the increasing proliferation of hand-held high-power laser pointers and their often reported misuse, their work is of high importance regarding the evaluation of performance limitations in the execution of human tasks in cases of laser dazzle.

For imaging sensors, we encounter similar considerations with respect to sensor performance when they get dazzled. At our institute, we have been working for many years on the protection of imaging sensors from laser threats, comprising sensor hardening against laser damage and laser dazzle [6]. In a recent publication, one of the authors (G. R.) presented an approach for laser safety calculations for imaging sensors [7]. The approach transforms the above-mentioned quantities of laser safety calculations for the human eye to imaging sensors. The equivalent quantities derived for imaging sensors were called maximum permissible exposure for a sensor (MPE_S_), nominal sensor hazard distance (NSeHD), maximum dazzle exposure for a sensor (MDE_S_) and nominal sensor dazzle distance (NSeDD). We refer the reader to the publication of Ritt for details on the derivation of these quantities [7].

Very briefly, the derivation is based on the estimation of the radial irradiance distribution of laser light at (or near) the focal plane of a camera lens. This estimation considers diffraction of light at the lens aperture and scattering of light from the surfaces of the optical elements. The estimated irradiance distribution is then compared to threshold values for laser dazzle or laser damage of the sensor to estimate the already mentioned laser safety quantities. The primary goal of this approach was to establish closed-form expressions containing only basic operations and functions in order to calculate such quantities. Furthermore, to perform such calculations mainly the standard parameters of such devices are required, specified by the manufacturers of laser sources, camera lenses and cameras/imaging sensors. However, in general manufacturers do not provide information on the scattering characteristics of their lenses. That makes it difficult to estimate the fraction of light scattering in the irradiance distribution at the focal plane. Typically, three parameters are sufficient to describe light scattering of optical elements. In reference [7], results of initial scatter measurements were presented for commercial off-the-shelf (COTS) camera lenses. However, the main issue behind the problems touches the question of whether there is a strong variation of these parameters from camera lens to camera lens or may a single set of scatter parameters be sufficient to generally describe the behavior of light scattering inside COTS camera lenses. The answer has a significant impact on the ease applicability of our concept regarding laser safety calculations for imaging sensors.

Dedicated literature that would help to solve our tasks could not be found. There is a vast amount of publications on stray light. Typical values of scatter parameters of optical elements (mirrors or lenses) are stated in text books (e.g., reference [8]) or overview articles (e.g., reference [9]). Furthermore, there are many journal articles dedicated to stray light analysis of optical systems using optical engineering software like TracePro, FRED or ASAP. A larger number of them is related to astronomical optical systems, like mirror telescopes. There seems to be only a lower number of publications that is related to pure refractive optical systems, e.g., the camera lens of cellphones [10] or infrared imaging systems [11].

Usually, the stray light analysis using an optical engineering software requires the statement of scatter parameters or, alternatively, the default values implemented in the software can be taken. In all the publications we found, only standard values for the scatter parameters were applied. We did not find a single publication, where scatter parameters for the optical system to be simulated were measured before. Therefore, this situation forced us to make our own efforts to estimate scatter parameters of COTS camera lenses, as input to our theoretical model.

In this publication, we report on our measurements of the irradiance distribution in the focal plane of COTS camera lenses when illuminated with laser light. The results have been used to estimate the scatter parameters required by the theoretical model of reference [7]. In Section 2, a brief review on this theoretical model is given and explained by means of calculations of the focal plane irradiance of a standard camera. Section 3 and Section 4 describe our experimental setup for the measurements and the camera lenses under test, respectively. Section 5 explains in detail the data analysis procedure for the estimation of the scatter parameters; the results are presented in Section 6. Finally, Section 7 treats with the simulation of stray light in camera lenses using the optical engineering software FRED in order to compare the theoretical model with the outcome of the FRED simulations.

## 2. Theoretical Background

### 2.1. Estimation of the Focal Plane Irradiance

**Figure 1:**
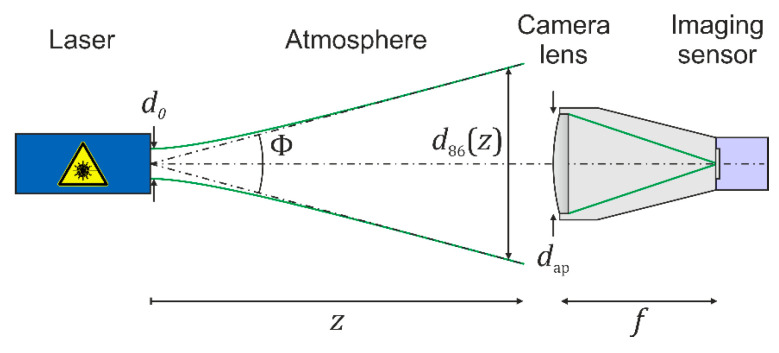
Schematic view of a dazzling scenario. A simple one-element lens represents the usually complex camera lens.

The theoretical model of publication [7] assumes a scenario as depicted in Figure 1. A laser emits a beam with a Gaussian beam profile and illuminates a sensor consisting of a camera lens and an imaging sensor. In Figure 1, the camera lens is represented by a single lens, but is treated as an optical system consisting of several optical elements. In order to describe the irradiance distribution at the focal plane of the camera lens and the response of the imaging sensor to the laser radiation, we used the parameters listed in Table 1.

Throughout this publication, we used the convention that the diameter of the laser beam is always the one at the position of the entrance aperture of the camera lens. Table 1 lists two definitions for the beam diameter. The first one, $d_{63}$ (m), is related to those points, where the irradiance has dropped to 1/e of the maximum irradiance. For Gaussian beams, 63 percent of the laser power is encircled within this diameter. When performing laser safety calculations, the use of the $d_{63}$-diameter is mandatory. The second (more common) definition, $d_{86}$ (m), is related to those points, where the irradiance has dropped to 1/e^2^ of the maximum irradiance. For Gaussian beams, 86 percent of the laser power is encircled within this diameter. The ratio of beam diameter $d_{86}$ to the diameter of the camera lens’ aperture (more precisely it is the entrance pupil) $d_{\mathrm{ap}}$ (m) is called the truncation factor ν and has a determining influence on the distribution of the laser light in the focal plane of the camera lens.

According to our theoretical model, the irradiance $E_{\mathrm{fp}}$ (W/m^2^) at the focal plane of a camera lens can be described by the sum of diffracted and scattered fractions of the incident light:

(1) $$E_{\mathrm{fp}}\left(r\right)=\eta_{d}E_{d}\left(r\right)+E_{s}\left(r\right)$$

This equation is identical to Equation (40) of reference [7] except that we here used the radial coordinate r (m) instead of the viewing angle Θ=r/f (rad). On the right-hand side of Equation (1), the three quantities $\eta_{d}$, $E_{d}\left(r\right)$ and $E_{s}\left(r\right)$ are given by Equations (39), (23) and (35a) of reference [7], respectively. There you also find details on the derivation of these equations. Briefly, $\eta_{d}$ describes the fraction of diffracted laser power. $E_{d}\left(r\right)$ is the irradiance distribution at the focal plane due to diffraction of a (truncated) Gaussian beam and $E_{s}\left(r\right)$ is the irradiance distribution at the focal plane due to light scattering from the surfaces of the various optical elements.

The term $E_{d}\left(r\right)$ is given by

(2) $$E_{d}\left(r\right)=\{\begin{array}{cc} E_{\mathrm{GA}}\left(r\right), & \;\left|r\right|\leq r_{\mathrm{pi}}\\ E_{\mathrm{mean}}\left(r\right), & \;\left|r\right|>r_{\mathrm{pi}} \end{array},$$

where $E_{\mathrm{GA}}\left(r\right)$ represents the central lobe of the diffraction pattern approximated by a Gaussian curve. Since for typical camera/lens configurations, the aperture caused diffraction ring pattern is usually not fully resolved, $E_{\mathrm{mean}}\left(r\right)$ describes the local mean irradiance of the wing parts of the diffraction pattern:

(3) $$E_{\mathrm{GA}}\left(r\right)=E_{0}\left(\nu \right)\exp\left(-8\frac{r^{2}}{d_{\mathrm{spot}}^{2}}\right)$$

(4) $$E_{\mathrm{mean}}\left(r\right)=\frac{P_{\mathrm{in}}T\lambda F}{\pi^{3}r^{3}}\cdot \frac{\frac{2}{\nu^{2}}\exp\left(-\frac{2}{\nu^{2}}\right)}{1-\exp\left(-\frac{2}{\nu^{2}}\right)}=\frac{P_{\mathrm{laser}}T\lambda F}{\pi^{3}r^{3}}\cdot \frac{2}{\nu^{2}}\exp\left(-\frac{2}{\nu^{2}}\right)$$

The radial coordinate $r_{\mathrm{pi}}$, which separates the central lobe from the wing parts of the diffraction pattern, cannot be stated in the analytical form but has to be calculated numerically; see Equation (22) of reference [7].

How much of the incident radiation will be diffracted is determined by the term $\eta_{d}$:

(5) $$\eta_{d}={\left(1-TIS\right)}^{N_{\mathrm{ss}}},$$

where $N_{\mathrm{ss}}$ is the number of scattering surfaces of the camera lens. TIS is the amount of total integrated scatter generated by one scattering surface [9]:

(6) $$TIS=\{\begin{array}{cc} 2\pi b\frac{100^{s}}{s+2}\;\left[{\left(1+l^{2}\right)}^{\frac{s+2}{2}}-{\left(l^{2}\right)}^{\frac{s+2}{2}}\right], & \;s\neq -2\\ 2\pi b\frac{{\left(100l\right)}^{s}}{2}l^{2}ln\left(1+\frac{1}{l^{2}}\right), & \;s=-2 \end{array}$$

This quantity is a function of the three scatter parameters s, b (sr^−1^) and l (rad). These scatter parameters originate from the 3-parameter Harvey scatter model (see below) used to describe light scattering caused by the surface roughness of optical elements.

The Harvey scatter model served Peterson to derive analytical equations to describe the distribution of scattered light in the focal plane of an optical system [13]. According to Peterson’s model, the stray light irradiance at the focal plane $E_{s}\left(r\right)$ can be estimated by adding up the contributions $E_{s,j}\left(r\right)$ of the single scattering elements:

(7) $$E_{s}\left(r\right)=\sum_{j=1}^{N_{\mathrm{ss}}}E_{s,j}\left(r\right),$$

where the contribution of a single scattering element is given by

(8) $$E_{s,j}\left(r\right)=\pi T{\left(NA\right)}^{2}\frac{a_{\mathrm{ent}}^{2}}{a_{j}^{2}}b_{0}{\left[1+{\left(\frac{\left(NA\right)r}{la_{j}}\right)}^{2}\right]}^{s/2}E_{\mathrm{ent}}.$$

Here, NA is the numerical aperture, $a_{\mathrm{ent}}$ (m) the radius of the beam at the first scattering element and $a_{j}$ (m) the radius of the beam at the jth scattering element. $E_{\mathrm{ent}}$ (W/m^2^) is the entering irradiance and $b_{0}$ is given by

(9) $$b_{0}=b\;{\left(100\cdot l\right)}^{s}.$$

We applied the following simplification and approximation on Peterson’s equations:

(10) $$a_{j}∶=a_{\mathrm{ent}}=\{\begin{array}{cc} \frac{d_{63}}{2}, & \;d_{63}<d_{\mathrm{ap}}\\ \frac{d_{\mathrm{ap}}}{2}, & \;d_{63}\geq d_{\mathrm{ap}} \end{array},$$

Thus, we got rid of the dependence on the beam radius $a_{j}$ in Equation (8) and, after some transformations (for details see reference [7]), we obtained a simpler equation for the scattering caused irradiance distribution $E_{s}\left(r\right)$ in the focal plane of a camera lens due to scattering of laser light:

(11) $$\begin{array}{c} E_{s}\left(r\right)=\frac{P_{\mathrm{in}}TN_{\mathrm{ss}}b_{0}}{f^{2}}\frac{1}{{\left(v^{*}\right)}^{2}}{\left[1+\frac{1}{{\left(v^{*}\right)}^{2}}\cdot {\left(\frac{r}{lf}\right)}^{2}\right]}^{\frac{s}{2}}\\ =\frac{P_{\mathrm{laser}}TN_{\mathrm{ss}}b_{0}}{f^{2}}\frac{1}{{\left(v^{*}\right)}^{2}}{\left[1+\frac{1}{{\left(v^{*}\right)}^{2}}{\left(\frac{r}{lf}\right)}^{2}\right]}^{\frac{s}{2}}\cdot \left(1-\exp\left(-\frac{2}{\nu^{2}}\right)\right) \end{array}$$

where $\nu^{*}$ is defined by

(12) $$\nu^{*}=\min\left(1,\frac{\nu }{\sqrt{2}}\right).$$

**Figure 2:**
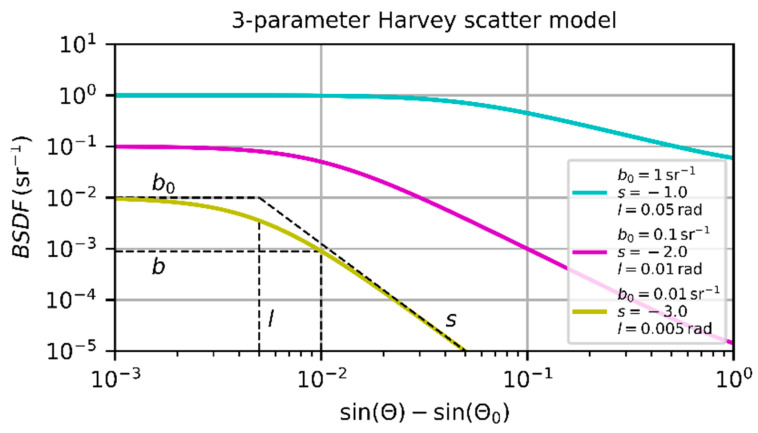
Bidirectional scattering distribution function according to the 3-parameter Harvey scatter model for different sets of scatter parameters.

Remark: Equation (11) corresponds to the form of the bidirectional scattering distribution function (BSDF) of the 3-parameter Harvey scatter model [8], expressed by

(13) $$BSDF=b_{0}{\left[1+{\left(\frac{\sin\left(\Theta \right)-\sin\left(\Theta_{0}\right)}{l}\right)}^{2}\right]}^{\frac{s}{2}}$$

when applying an incidence angle of $\Theta_{0}=0$ and using the small angle approximation sin(Θ)≈Θ. Choosing three different sets of scatter parameters $b_{0}$, s and l, Figure 2 shows their influence on the BSDF. At low scatter angles Θ, the scattering signal stays constant and decreases with increasing values of Θ. In a double-logarithmic plot, the decay appears linear. The meaning of the three scatter parameters is depicted using the yellow curve in in Figure 2. Scatter parameter l indicates the scatter angle where the BSDF changes from the constant region to the decreasing part of the curve with slope s. In a double-logarithmic plot and for scatter angles Θ≫l, the scatter parameter s represents the slope of the BSDF. Scatter parameters b is the value of the BSDF for $\sin\left(\Theta \right)-\sin\left(\Theta_{0}\right)=0.01$ and scatter parameter $b_{0}$ describes the maximum BSDF value.

Generally, the scatter parameters are material parameters with fixed values but they are supposed to change with wavelength. The wavelength scaling laws are given by [14]

(14) $$\begin{array}{c} b\left(\lambda \right)=b\left(\lambda_{0}\right){\left(\frac{\lambda_{0}}{\lambda }\right)}^{4+s}\\ s\left(\lambda \right)=s\left(\lambda_{0}\right)\\ l\left(\lambda \right)=l\left(\lambda_{0}\right)\left(\frac{\lambda }{\lambda_{0}}\right) \end{array}$$

with $\lambda_{0}$ as the reference wavelength, for which the scatter parameters are known, and λ is the wavelength, for which the scatter parameters shall be calculated.

Based on the equations described here, we can see that our theoretical model to estimate the focal plane irradiance distribution for camera lenses depends mainly on standard parameters of the devices, besides the three scatter parameters s, b, l and the truncation factor ν.

### 2.2. Estimation of the Camera Response

The output of a digital camera is an image consisting of a number of pixels comprising digital gray values. Since we want to compare such digital images with the theoretical model described before in the further course of this publication, we need to transfer these digital values to irradiance values (or the other way round). For a camera that has a linear response to the number of photons arriving during exposure time, the digital signal can be calculated according to the EMVA 1288 standard by [15]

(15) $$\mu_{y}=K\cdot \eta \cdot \frac{EAt_{\exp}}{\frac{hc}{\lambda }}+\mu_{y.\mathrm{dark}},$$

where E (W/m^2^) is the irradiance at the pixel, A (m^2^) the pixel area, $t_{\exp}$ (s) the camera’s exposure time, $h=6.626\cdot 10^{-34}\;\mathrm{Js}$ the Planck constant, $c=2.99792458\cdot 10^{8}\;m/s$ the vacuum speed of light, λ (m) the wavelength, η the quantum efficiency, K (DN/e-) the overall system gain and $\mu_{y.\mathrm{dark}}$ (DN) the dark signal.

**Figure 3:**
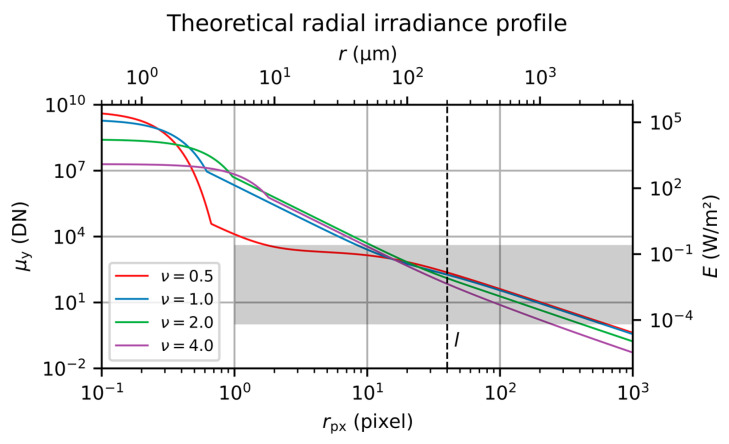
Theoretical radial irradiance profile for different values of truncation factor ν.

As an example, Figure 3 shows a set of four curves for the camera signal $\mu_{y}$ and irradiance E as a function of radial coordinate r for different values of the truncation factor ν. The curves were calculated using Equations (1) and (15) with the following set of parameters:

- Laser: $P_{\mathrm{laser}}=1\;\mu W$, λ=532 nm, $d_{86}=25\;\mathrm{mm}$;
- Camera: p=5 μm, η=0.6, $t_{\exp}=1\;\mathrm{ms}$, $K=0.4\;\mathrm{DN}/e^{-}$, bd=12 bit, $\mu_{y.\mathrm{dark}}=0$;
- Camera lens: f=100 mm, F={2,4,8,16}, $N_{\mathrm{oe}}=7$, T=0.93, s=−2, b=0.4, l=2 mrad.

For the values of the scatter parameters, we anticipated mostly the results of our measurements presented in Section 6. The scatter parameter l=2 mrad corresponds to a radial coordinate of $r_{l}=l\cdot f=200\;\mu m$ or $r_{l}/p=40\;\mathrm{pixel}$, which is marked by a dashed vertical line in Figure 3. The set of different f-numbers F results in a set of different truncation factors $\nu =d_{86}/\left(f/F\right)$ of 0.5, 1.0, 2.0 and 4.0. Since the emitted laser power was kept constant for the calculation, the incident power $P_{\mathrm{in}}$ entering the camera is different for each plotted curve and was calculated using the equation given in Table 1.

The camera signal $\mu_{y}$ as plotted in the graph does not correspond to a real camera. A real camera is only able to generate signals within a certain dynamic range, marked in the graph by a gray background covering radial coordinates $r_{\mathrm{px}}\geq 1$ pixel and signals of $\mu_{y}\in \left[1\;\mathrm{DN},2^{bd}-1\;\mathrm{DN}\right]$. For our example camera, signals $\mu_{y}$ above the upper limit of $2^{12}-1\;\mathrm{DN}=4095\;\mathrm{DN}$ would be restricted to this value, i.e., the camera would be saturated. Signals $\mu_{y}<1\;\mathrm{DN}$ could only be measured as an average signal of a multitude of pixels. For a real camera, the upper and lower usable limits are given by the saturation gray value $\mu_{y.\mathrm{sat}}$ (DN) and the absolute sensitivity threshold $\mu_{y.\min}$ (DN), respectively. These values are slightly different to the theoretical values of 4095 DN and 1 DN.

## 3. Experimental Setup and Measurement Procedure

**Figure 4:**
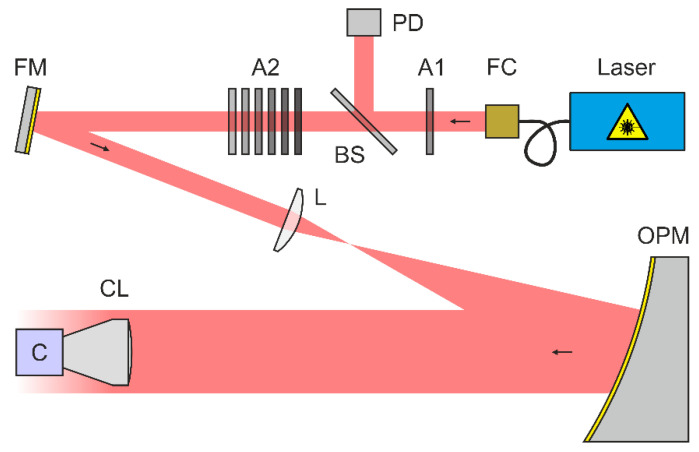
Scheme of the experimental setup for the measurement of irradiance distribution in the focal plane of camera lenses. FC: fiber collimator, A1/A2: attenuator, BS: beam splitter, PD: (reference) photodiode, FM: folding mirror, L: focusing lens, OPM: off-axis parabolic mirror, CL: camera lens, C: camera.

A scheme of our experimental setup to measure the irradiance at the focal plane of a camera lens is shown in Figure 4. As a light source, we used a Toptica iChrome MLE multi-wavelength laser source. This laser source offers four different laser wavelengths of 488 nm, 515 nm, 561 nm and 640 nm, which all were coupled into a common single-mode fiber. Depending on the wavelength, the output power at the fiber exit port ranged from 40 to 100 mW. The laser light was collimated using a reflective fiber collimator FC (Thorlabs RC08APC-P01). Attenuator A1 (neutral density filter Thorlabs NE40B-A) was used to set the maximum laser power to a value in the order of 1 µW. Subsequently, the light path was divided using beam splitter BS (Thorlabs CM1-BP1), sending the reflected part to the reference photodiode PD (Ophir PD300R-UV sensor head with power meter Ophir Vega). The transmitted light passed a second attenuator A2, consisting of a set of neutral density filters (Thorlabs NExxB, where xx relates to the optical density). In the further course, the laser beam passed a folding mirror FM and was expanded by a Keplerian telescope with magnification M=6.7 built by a focusing lens L (Thorlabs LA1484-A, $f_{1}=300\;\mathrm{mm}$) and an off-axis parabolic mirror OPM (Optical Surfaces Ltd. 037-0220, $f_{2}=2000\;\mathrm{mm}$). Finally, the collimated laser beam was sent to the camera lens CL under test. The laser beam diameters at the entrance of the camera lens were 21.5, 21.1, 20.3 and 20.1 mm for the laser wavelengths of 488, 515, 561 and 640 nm, respectively. For details on the laser beam diameter measurements see Appendix B.

The wavelength-dependent parameters of the optical elements (splitting ratio of the beam splitter BS, transmittance of the attenuator A2 and lens L, reflectivity of folding mirror FM and off-axis parabolic mirror OPM) were calibrated before the measurements. Therefore, we were able to calculate the power within the laser beam at the position of the camera lens CL using the reading of the reference photodiode PD.

In order to measure the stray light irradiance at the focal plane of the camera lens, we used camera C (Allied Vision Mako G-419B NIR, Stadtroda, Germany) as a detector. The parameters of the camera are given in Table 2, which lists parameters as specified by the manufacturer [16] and parameters measured by ourselves. The latter were measured according to the EMVA 1288 standard; see Appendix B. From Figure 3 we can see that the central lobe of the diffraction pattern is within a radius of about 1–2 pixels of our camera. Therefore, we could not expect to resolve the central lobe with our camera-based experimental setup.

Each camera lens under test (with attached camera C) was centered with respect to the optical axis defined by the laser beam. Thus, the center of the laser spot coincided mostly with the center of the detector. Since the tested camera lenses were different in length, each camera lens was positioned so that the front facet of the camera lens coincided with that position on the optical axis where the laser beam diameter was measured. The camera and the laser were switched on 30–60 min before performing the measurements to ensure thermal equilibrium conditions.

**Figure 5:**
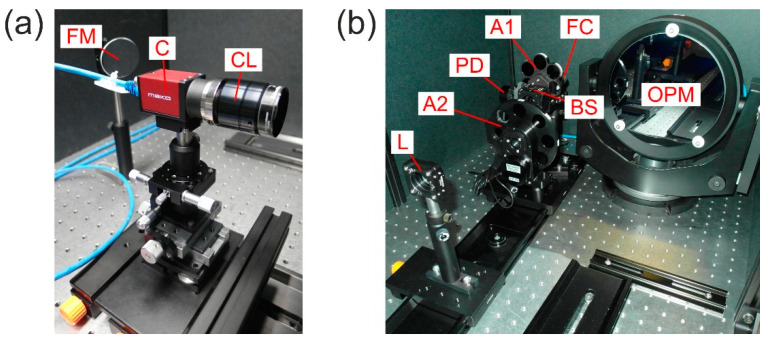
Photographs of different parts of the experimental setup: (**a**) camera C with camera lens CL and folding mirror FM and (**b**) fiber collimator FC, attenuators A1 and A2, beam splitter BS, (reference) photodiode PD, focusing lens L and off-axis parabolic mirror OPM.

Figure 5 shows some photographs of the experimental setup. The complete experimental setup was covered by a housing to prevent from ambient light. We also made sure that the residual light in the laboratory (e.g., due to the computer monitor, emergency exit lights, etc.) had no noticeable effect on the camera signal. All experimental parameters were controlled using a computer, whereby the lens’ f-number had to be set manually.

The main challenge in measuring the irradiance distribution of a focused laser beam is the high dynamic range of irradiance values, which have to be covered. At the center of the laser spot, the irradiance is quite high (10^4^ W/m^2^ in Figure 3), whereas the off-center stray light irradiance is quite low (10^−4^ W/m^2^ in Figure 3). Typically, the dynamic range of camera sensors is in the order of 60 dB, which means that the ratio of highest to lowest measureable irradiance corresponds to a factor of only 1000. This is by far not high enough to measure the irradiance within the whole area of the imaging sensor by capturing just one single image. Therefore, to gain an image with full intensity distribution, i.e., full dynamic range, we had to acquire a number of camera images based on different combinations of laser power and camera exposure time. For the further course of the publication, we introduced a naming convention for the different measurement steps, which are explained below in more detail; an overview is given in Table 3.

For each camera lens under test, we started the experiments by setting a specific f-number F. Using a selected setting of camera exposure time $t_{\exp}$ and laser power $P_{\mathrm{laser}}$, in the first step, we performed the image acquisition process. Dependent on the exposure time and laser power used, some parts of such an image may be overexposed or too noisy. Thus, such a single image will only deliver a part of the complete radial irradiance profile with a linear signal response. In order to get the desired irradiance information for almost the complete area of the imaging sensor, this image acquisition process was repeated seven times for different combinations of exposure time $t_{\exp}$ and laser power $P_{\mathrm{laser}}$ (see Table 4) to obtain a total of eight image acquisitions. We call this process of eight image acquisitions a measurement. Then, such a measurement performed for each of the four available laser wavelengths, results in a measurement series. By repeating such a measurement series for each labeled f-number F of a camera lens, we obtained a complete data set for this specific camera lens. The output of this procedure accomplished for all the mentioned camera lenses (see Section 4) eventually formed a data ensemble.

Besides the image acquisitions with laser illumination, we also acquired images without illumination (dark frames), necessary for the later data analysis. The dark frames were recorded according to the exposure times listed in Table 4.

**Figure 6:**
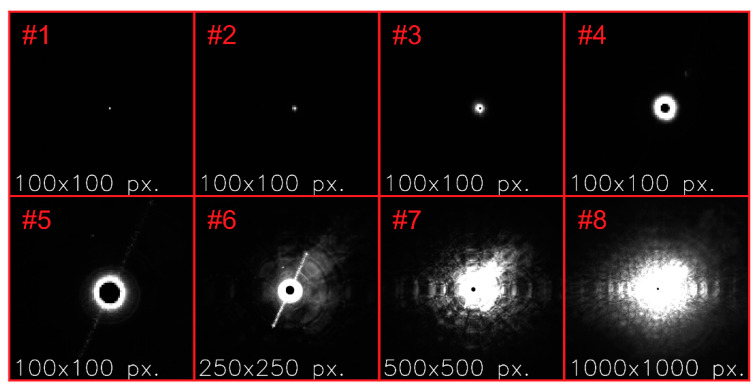
Examples of camera images acquired for different values of exposure time and laser power. The labels correspond to the setting numbers of Table 4. Camera lens: Edmund Optics 86410, experimental parameters: λ=488 nm, F=2.8.

Figure 6 shows some example images for the camera lens Edmund Optics 86410, acquired at the laser wavelength of λ=488 nm and for an f-number of F=2.8. The red labels correspond to the setting number indicated in Table 4. Please note, that the individual images do not show the complete camera image comprising 2048 pixels × 2048 pixels, but only the central section. The individual image sections also differed in size depending on the exposed area; see the white annotations within the images.

## 4. Camera Lenses Tested

Table 5 lists the lenses used for the experiments and their specifications. Intentionally, low-priced and higher-priced camera lenses were chosen: seven commercial off-the-shelf (COTS) camera lenses and one COTS achromatic doublet lens. The lenses differed in their values of focal length, f-number and number of optical elements.

**Figure 7:**
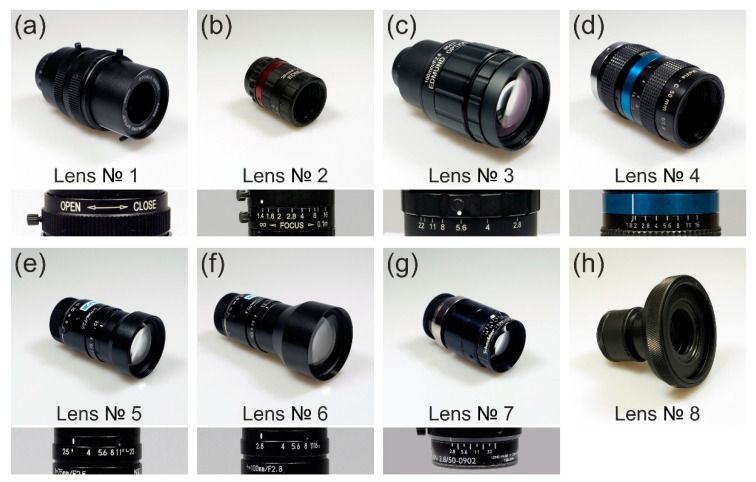
Camera lenses used for the experiments: (**a**) Edmund Optics 54690, (**b**) Edmund Optics 67715, (**c**) Edmund Optics 86410, (**d**) LINOS MeVis-C 1.8/50, (**e**) Navitar NMV-75, (**f**) Navitar NMV-100, (**g**) Schneider-Kreuznach Xenoplan 2.8/50 and (**h**) Thorlabs AC254-050-A (achromatic doublet).

The set of f-numbers used for the measurements varied for each camera lens. We only selected those settings that were labeled on the aperture ring. One camera lens (Edmund Optics 54690) had no labels at the aperture ring. Here, we only used the minimum and the maximum f-number (largest und smallest aperture) for the experiments. In case of the achromatic doublet Thorlabs AC254-050-A, we used an external iris (Thorlabs SM2D25D) directly in front of the lens to obtain different settings for the f-number. Figure 7 shows photographs of all lenses and in addition, details of their aperture rings. For the achromatic doublet, the image shows the complete assembly with tube housing and an external iris.

Table 6 lists the settings of the f-numbers used for the experiments and the corresponding mean truncation factors. Since the laser beam diameter slightly varied with the wavelength, the truncation factor also varied slightly with the wavelength and therefore only the mean truncation factors $\bar{v}$ are given.

## 5. Data Analysis

The aim of our investigations was to estimate for each camera lens the scatter parameters s, b and l, in order to generate a generic set of scatter parameters. In the course of our experiments, we analyzed eight different lenses (seven camera lenses and one achromatic doublet, see Section 4) applying four different laser wavelengths at various integration times and laser powers, while the laser beam diameter was kept constant throughout. For each lens, the data set contained 1–7 measurement series, depending on the number of labeled settings of the lens’ aperture ring. The data ensemble of eight data sets contained in total 43 measurement series (see Table 6), i.e., 43 × 4 = 172 measurements or 43 × 4 × 8 = 1376 camera images, to be analyzed. Since the amount of data was quite huge, we used automated analysis software to derive the scatter parameters. Briefly, this included the following steps:

1. Irradiance profile generation: For each acquired image, an irradiance profile was assessed from the image data.
2. Profile stitching: Due to the limited dynamic range of the camera, the merging of the eight radial irradiance profiles of a measurement was necessary to get a complete radial irradiance profile for the specific experimental set-up (camera lens under test, f-number F and laser wavelength λ).
3. Curve fitting: Fitting of a theoretical curve to the radial irradiance profiles of a measurement series (comprising the measurements with all four laser wavelengths) using the scatter parameters s, b and l as fitting parameters.

Now, we will describe in detail the above-mentioned analysis process. As an example, we used part of the data of the camera lens Edmund Optics 86410. Example images acquired with this camera lens for a laser wavelength of λ=488 nm and an f-number F=2.8 are shown in Figure 6. Subsequent to the data analysis process for all camera lenses, we performed a statistical analysis of the scatter parameters. These results are presented in Section 6.

### 5.1. Irradiance Profile Generation

#### 5.1.1. Dark Frame Correction

Before analyzing the image data, we performed dark frame corrections on the laser-illuminated images, i.e., the dark frames were subtracted from the laser-illuminated images to remove the dark signal. The residual signal of the camera pixels is then proportional to the incident irradiance; see the camera’s linearity in Appendix B.

#### 5.1.2. Estimation of the Center of the Laser Spot

As a prerequisite for the generation of the irradiance profile, the center of the laser spot within the camera images had to be determined first. Although the alignment of the camera (with attached camera lens) ensured that the position of the laser spot was always close to the center of the imaging sensor (see Section 3) the slight variations regarding the exact center could not be neglected.

To find the center of the laser spot the following procedure was applied: we chose the three images acquired at the lowest laser power, using settings no. 1–3 of Table 4. From each of these three images, we extracted the central part of 100 pixels × 100 pixels and calculated a mean image. Subsequently, the pixel with a maximum signal within this mean image (coordinates: column $x_{c}$, row $y_{c}$) was identified and defined as the center of the laser spot.

**Figure 8:**
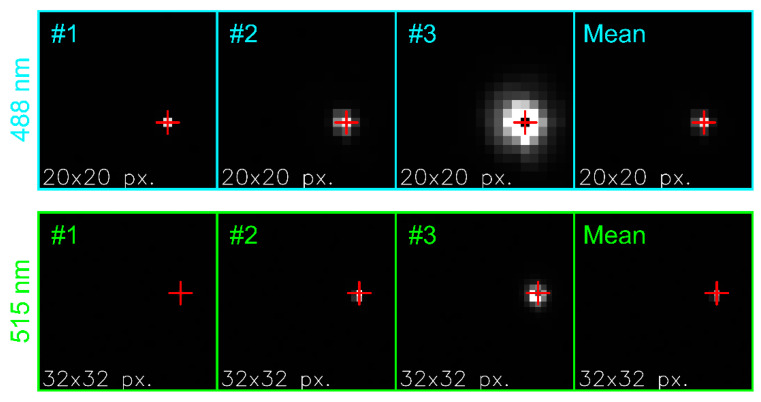
Estimation of the laser spot center for the camera lens Edmund Optics 86410 (F=2.8). Top row: λ=488 nm, bottom row: λ=515 nm. From left to right: Sections of camera images acquired with setting no. 1–3 of Table 4 and the corresponding mean image. A red cross marks the center of the laser spot (estimated from the mean image). The red crosses in the images #1–#3 are derived from the mean image.

For the camera lens Edmund Optics 86410 (F=2.8) this procedure is depicted in Figure 8 for the two laser wavelengths of 488 nm and 515 nm. In each frame, a red cross marks the estimated center of the laser spot.

#### 5.1.3. Estimation of the Radial Irradiance Profile

**Figure 9:**
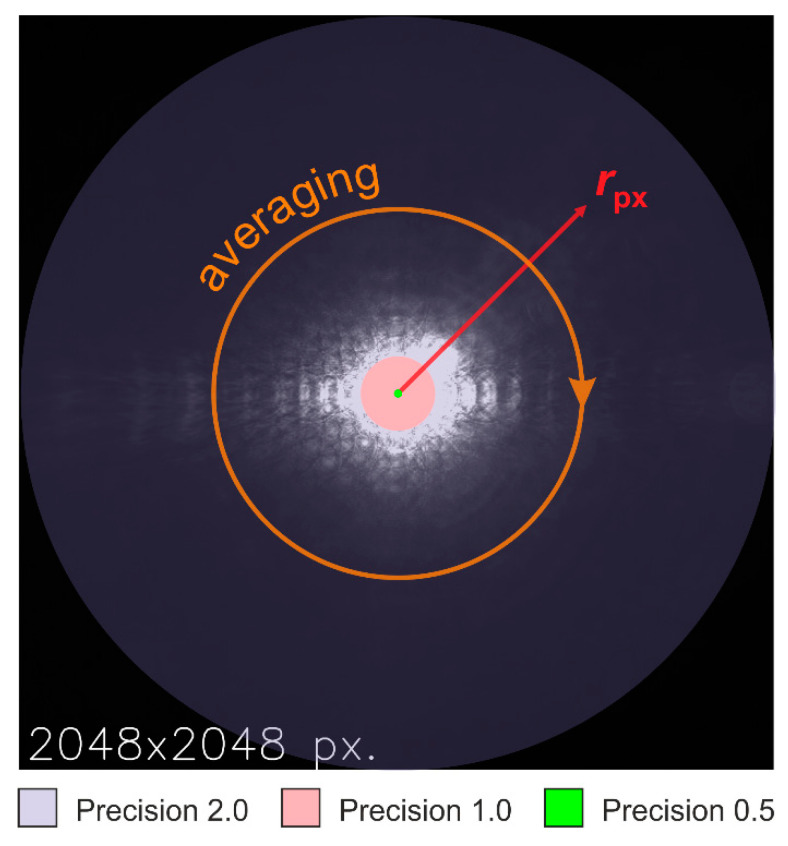
Illustration of the averaging process. The signals of all those pixels having the same distance $r_{\mathrm{px}}$ (red coordinate axis) to the laser spot center are averaged (depicted by the circular arrow in orange color) in order to find the radial irradiance distribution. Colored disks, overlaid to the camera image, depict areas where different rounding precisions were applied. The camera image was taken using the lens Edmund Optics 86410 (λ=488 nm, F=2.8, setting no. 8).

In order to find the lens-generated radial irradiance profile in the focal plane, the pixel values for each occurring radial distance r to the center of the laser sport were averaged. The principle of the process is illustrated in Figure 9. It shows a complete image of 2048 pixels × 2048 pixels, as taken with setting no. 8 in Figure 6. A red arrow indicates the radial coordinate axis. The orange arrow depicts the circular averaging process for a specific radial coordinate.

The averaging process first demands to calculate the radial distance $r_{\mathrm{px}}$ (pixel) of each pixel in the frame regarding the center of the laser spot $\left(x_{c},y_{c}\right)$:

(16) $$r_{\mathrm{px}}=\left|\sqrt{{\left(x-x_{c}\right)}^{2}+{\left(y-y_{c}\right)}^{2}}\right|$$

As one can easily see, for each occurring value of distance $r_{\mathrm{px}}$ there are only eight pixels with exactly the same distance $r_{\mathrm{px}}$ to the center of the laser spot. This would have led to a set of data points for the irradiance profile with a large number of values for the independent variable $r_{\mathrm{px}}$, but a low statistical basis for the dependent variable $\mu_{y}$. Therefore, before averaging took place the values of $r_{\mathrm{px}}$ were rounded. The precision for rounding was adapted to different ranges of the radial coordinate:

- Rounding precision: 0.5 for $r_{\mathrm{px}}<10$;
- Rounding precision: 1.0 for $10\leq r_{\mathrm{px}}<200$;
- Rounding precision: 2.0 for $r_{\mathrm{px}}\geq 200$.

The areas with different rounding precision are marked in Figure 9 by colored disks, overlaid to the camera image. The rationale behind this choice of these areas will become obvious in Section 5.3.1.

**Figure 10:**
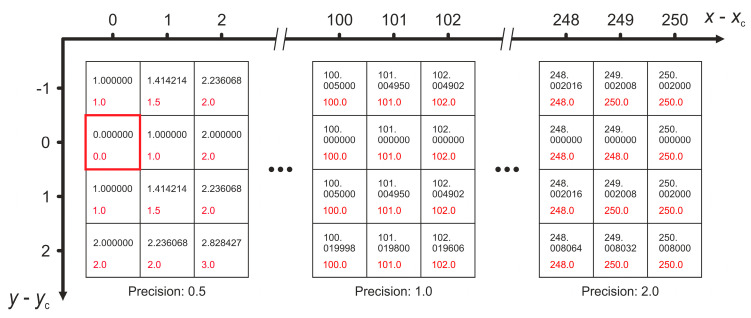
Rounding process for the values of the radial coordinate $r_{\mathrm{px}}$. Black numbers: exact values of $r_{\mathrm{px}}$ and red numbers: rounded values of $r_{\mathrm{px}}$.

The rounding procedure is illustrated in Figure 10 for better understanding. The squares represent individual pixels out of the 2048 pixels × 2048 pixels of the imaging sensor, whereby the three pixel blocks represent three distant regions where the rounding precision was adapted to an individually chosen value, i.e., rounding precision of 0.5, 1.0 and 2.0. The coordinate axes indicate the x/y-coordinates of the pixels regarding the laser spot center $\left(x_{c},y_{c}\right)$, which is highlighted by the red frame in the left pixel block. Inside the squares, the black numbers show the radial coordinate $r_{\mathrm{px}}$, exactly calculated to six decimal places while the red numbers show the corresponding rounded value of $r_{\mathrm{px}}$, used for later analysis according to the above-described procedure.

**Figure 11:**
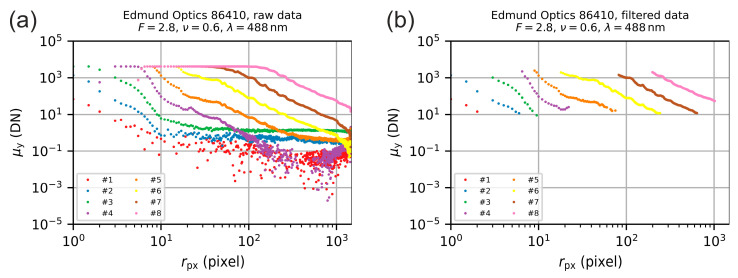
Radial irradiance profile for the camera lens Edmund Optics 86410 (λ=488 nm, F=2.8). (**a**) Extracted radial irradiance profiles for the different settings according to Table 4. (**b**) Remaining radial irradiance profiles after discarding values of overexposed and underexposed parts of the images.

For an acquired image, the successive circular averaging processes resulted in a radial irradiance profile. According to this procedure, the image data of Figure 6 was treated correspondingly and the respective radial irradiance profiles are shown in Figure 11a. The results shown there, we denote as raw data, since the curves contain also measurement values not usable for the further processing.

From Figure 11a, we can clearly recognize the saturated parts of the various irradiance profiles (course of the curves is horizontal) and parts that look noisy (strong signal scattering). Therefore, we filtered the data and kept only those values that belong to the slope of the curves:

1. We dismissed those radial coordinates for which
  - More than 10 percent of the pixels have gray values larger than the saturation gray value $\mu_{y.\mathrm{sat}}$ (DN) or;
  - More than 10 percent of the pixels have gray values lower than the absolute sensitivity threshold $\mu_{y.\min}$ (DN).
2. For the signals of those radial coordinates that were not dismissed, we calculated the average using only those pixel values that were within the limits $\left[\mu_{y.\min},\mu_{y.\mathrm{sat}}\right]$, i.e., we calculated a trimmed mean.

The saturation gray value of our camera was estimated to be $\mu_{y.\mathrm{sat}}=3861$; see Appendix B. The absolute sensitivity threshold $\mu_{y.\min}$ (related to the digital gray value) was calculated from the corresponding value $\mu_{e.\min}$ (related to the number of photoelectrons) by

(17) $$\mu_{y.\min}=K\cdot \mu_{e.\min},$$

where K (DN/e-) is the overall system gain. For our camera, the absolute sensitivity threshold was $\mu_{e.\min}=14.1\;e^{-}$ and the overall system gain was $K=0.399\;\mathrm{DN}/e^{-}$ (see Table 2). Thus, for the sensitivity threshold we get $\mu_{y.\min}=0.399\;\mathrm{DN}/e^{-}\;\cdot 14.1\;e^{-}\approx 6\;\mathrm{DN}$.

Furthermore, we only kept that part of the irradiance profile, where the radial coordinates do not exceed the edges of the camera image in any direction. This means the camera signals in the corners of the image were discarded. The maximum value of $r_{\mathrm{px}}$ is depicted in Figure 9 by the outer edge of the blue disk for a rounding precision of 2.0. For a perfect alignment of the laser spot to the exact center of the imaging sensor, the maximum value of $r_{\mathrm{px}}$ would correspond to 1023 pixels. The residual data after the complete filtering process is shown in Figure 11b.

### 5.2. Profile Stitching

**Figure 12:**
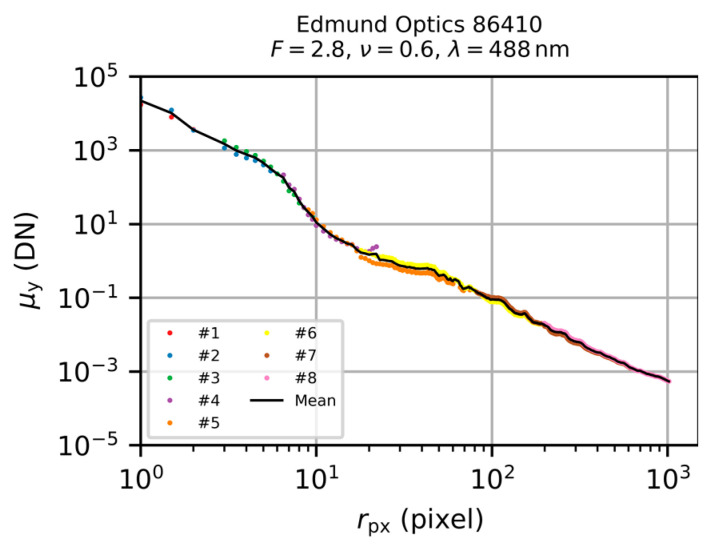
Scaled radial irradiance profile section of Figure 11b by taking into account the differently set parameters of the camera’s exposure time, the optical density of attenuator A2 and the laser power during image acquisition.

To get the complete radial irradiance profile, the individual profile sections were stitched together. Since they were derived for different settings of the camera’s exposure time and the optical density of attenuator A2, we had to correct the values accordingly. For this, we scaled the values by a factor

$${\left(\frac{t_{\exp,i}}{1\;µs}\;\cdot 10^{-OD_{A2,i}}\cdot \frac{P_{i}}{\bar{P}}\right)}^{-1},$$

where i is the setting number according to Table 4, $P_{i}$ the laser power during image acquisition for setting number i and $\bar{P}=\sum_{i=1}^{8}P_{i}/8$ is the mean power during the whole measurement. The individual radial irradiance profile sections of Figure 11b scaled in this way are shown in Figure 12. Since the individual profiles may overlap regarding the radial coordinate r, we additionally calculated the mean of those values having the same radial coordinate. The resulting complete irradiance profile is shown in Figure 12 by the black solid line and was used for the further analysis process. It would correspond to a profile a sensor would provide when there were no limitations in its dynamic range and using an exposure time of 1 µs.

### 5.3. Curve Fitting

Subsequent to the estimation of the complete radial irradiance profiles, different curves based on different theoretical models were fitted to the data in order to find the desired scatter parameters. Here, we described our approach and the rationales behind it.

For fitting the data, we used three different theoretical models, which we will discuss at a later stage in detail:

- Model M1: Our original theoretical model according to Equation (1).
- Model M2: An extension of model M1 using an additional empirical term to describe the effects of aberrations.
- Model M3: A simplification of model M1.

All models have in common that they describe the spatial distribution of the irradiance E in the focal plane of a camera lens as function of the radial coordinate r (m) and assuming rotational symmetry. That makes it necessary to adapt the equations of the corresponding models to the irradiance profiles that represent the camera signal $\mu_{y}$ in units of digital numbers (DN) as function of the radial coordinate $r_{\mathrm{px}}$ (pixel). For this, we related the radial coordinate r in our theoretical models to the radial coordinate $r_{\mathrm{px}}$ by

(18) $$r=r_{\mathrm{px}}\cdot p,$$

where p (m) is the pixel size of the camera (p=5.5 μm). Furthermore, the irradiance values had to be transformed into camera signals using Equation (15) with $\mu_{y.\mathrm{dark}}=0$, because of the dark frame correction:

(19) $$\mu_{y}=K\cdot \eta \cdot \frac{A\;t_{\exp}\;E}{\frac{hc}{\lambda }}.$$

Due to the profile stitching process, see Section 5.2, we used a fixed exposure time of $t_{\exp}=1\;\mu s$.

#### 5.3.1. Model M1: Our Original Model

In a first step, we used our original theoretical model M1 to simulate the focal plane irradiance as described by Equation (1):

(20) $$E_{M1}\left(r\right)=E_{\mathrm{fp}}\left(r\right)=\eta_{d}E_{d}\left(r\right)+E_{s}\left(r\right)$$

The scatter parameters s, b and l of the term $E_{s}\left(r\right)$ were used as fit parameters. The fitting ranges for these parameters were [−3.5≤s≤−0.5], [0.01≤b≤100] and $\left[5\cdot 10^{-4}\leq l\leq 0.1\right]$.

In principle, we could perform a fit on the data of each measurement (single wavelength). Alternatively, we could perform a fit on the complete data of the measurement series (comprising all four laser wavelengths) by including the wavelength-scaling laws for the scatter parameters given by Equation (14). We recognized that the latter method leads to a more robust fit and, thus, used this method for the data analyses. The outcome of the fitting process was always related to a reference wavelength of 550 nm.

**Figure 13:**
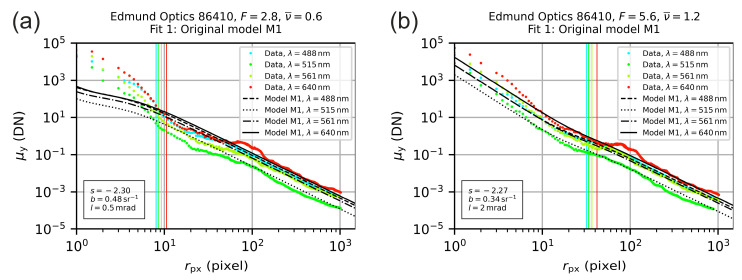
Radial irradiance distribution for the lens Edmund Optics 86410 operated with an f-number of (**a**) F=2.8 and (**b**) F=5.6. The colored points represent the measured values. The lines are the result of fitting the theoretical model M1 to the data. The vertical lines indicate the values of the respective scatter parameters l.

As an example for the curve fitting according to model M1, Figure 13 shows the derived irradiance profiles for the camera lens Edmund Optic 86410 for two different values of the f-number; Figure 13a F=2.8 and Figure 13b F=5.6. The irradiance profiles are plotted as colored points, where the color corresponds to the laser wavelength. The curve of Figure 12 (λ=488 nm) can be found as blue data points in Figure 13a. The black lines show the model curves after the fitting process. The value of the scatter parameter l is indicated by the vertical lines; the color coding of these lines corresponds to that of the data points.

By the example of Figure 13, we can see that the radial range of $10\;\mathrm{pixel}<r_{\mathrm{px}}<200\;\mathrm{pixel}$ represents a transition range, which divides the region where mainly scatter dominates ($r_{\mathrm{px}}>200\;\mathrm{pixel}$) from the region where mainly diffraction (and aberration) dominates ($r_{\mathrm{px}}<10\;\mathrm{pixel}$). This transition is much more pronounced for smaller values of the truncation factor ν. For very high values of the f-number, the transition may even not be clearly visible. These observations apply to all the camera lenses and are the reason for our choice to apply different values of rounding precisions for the profile generation process described in Section 5.1. Most of the data is located in the region $r_{\mathrm{px}}>200\;\mathrm{pixel}$. By our choice of the rounding precisions, we were able to adjust the number of data points in such a way that the region $r_{\mathrm{px}}>200\;\mathrm{pixel}$ does not dominate the fitting result and that the characteristics of the curve for $r_{\mathrm{px}}<200\;\mathrm{pixel}$ also influence the outcome of the curve fit.

Furthermore, we can see that there is a discrepancy between the irradiance profile and the model for very small radial coordinates ($r_{\mathrm{px}}<10\;\mathrm{pixel}$). We attributed this to the fact that model M1, described by Equation (1), did not include aberrations. However, aberrations will affect the center of the laser spot. The observed discrepancy is stronger for small values of truncation factor ν. This discrepancy leads to strong variations of the fitting results for different values of the f-number; see, e.g., the different location of the vertical lines in Figure 13a,b.

For this reason, we adapted our analysis process and extended model M1 by an additional term (then named model M2) in order to minimize the deviations between the theoretical model and the measured data in the vicinity of the center of the laser spot.

#### 5.3.2. Model M2: The Auxiliary Model

By extending model M1 by an empirical additional term, we got model M2, which may be seen as an approach to account for the previous discrepancies between model M1 and the measured data at the center of the laser spot:

(21) $$E_{M2}=\left(p_{1}\cdot \exp\left(-\frac{r^{2}}{2p_{2}^{2}}\right)+1\right)\cdot \eta_{d}E_{d}\left(r\right)+E_{s}\left(r\right)$$

**Figure 14:**
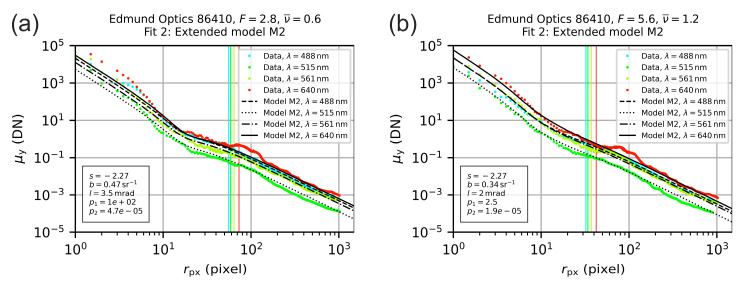
Radial irradiance distribution for the lens Edmund Optics 86410 operated with an f-number of (**a**) F=2.8 and (**b**) F=5.6. The colored points are the measured values derived from the analysis of the camera images. The lines are the result of fitting the theoretical model M2 to the data.

The additional term (red part in Equation (21)) extends the diffraction term $\eta_{d}E_{d}\left(r\right)$ for aberrations by multiplying it with a Gaussian function. Mathematically, the two parameters $p_{1}$ and $p_{2}$ describe the amplitude and the width of the Gaussian function, respectively, and were used as additional fitting parameters. The fitting parameters $p_{1}$ and $p_{2}$ were kept constant for all laser wavelengths. The auxiliary model M2 is able to describe the measurement results much better near the center of the laser spot as compared to the original model M1. Figure 14 presents the results achieved with model M2 for the same examples as given in Figure 13.

**Figure 15:**
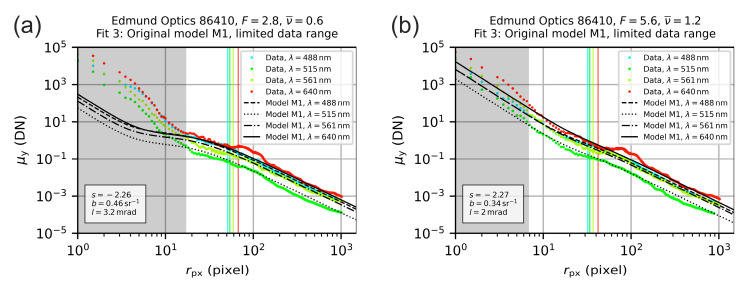
Radial irradiance distribution for the lens Edmund Optics 86410 operated with an f-number of (**a**) F=2.8 and (**b**) F=5.6. The colored points are the measured values derived from the analysis of the camera images. The black lines are the result of fitting the theoretical model M1 to the data within a limited range of the radial coordinate (excluded data is shaded in gray).

Since we had no real physical explanation for the new parameters $p_{1}$ and $p_{2}$ in the auxiliary model, we just used the fitting parameter $p_{2}$ to set a lower limit of the radial coordinate r to perform alternative curve fits with model M1, but now based on an adjusted radial coordinate range. We defined the range where the measurement values deviated from the theoretical model M1 as $\left[0,\;2p_{2}\right]$ and set the minimum value of the radial coordinate to $r_{\min}=2p_{2}$ for the second fitting process with model M1. Results are presented in Figure 15 for the same examples as used before, where the excluded data is indicated in the plots by a gray background. We can see that now the location of the vertical lines is comparable for the two different f-numbers.

#### 5.3.3. Model M3: The Simplified Model

The derivation of the scatter component $E_{s}\left(r\right)$ of our original model M1 required taking the diameter of the incident light beam at the optics into account [7]. Two cases had to be distinguished, see Equation (10): (i) For $d_{63}$-beam diameters smaller than the lens aperture $d_{ap}$ we used $d_{63}$ as the beam diameter for the calculations. (ii) For beam diameters $d_{63}$ larger than the lens aperture $d_{ap}$, the beam diameter was set to the value of the lens aperture $d_{ap}$. This definition led to the factor $\nu^{*}$ of Equation (12). Now, as an assumption, we set $\nu^{⋆}≔1$. This means that the incident light beam is assumed to fill the complete lens aperture. For Gaussian laser beams, this assumption is not false since the wings of a Gaussian beam profile extends, at least in theory, to infinity. Such an assumption would simplify the model M1. In the course of our studies, we assessed this approach, leading to a simplified model M3:

(22) $$E_{M3}\left(r\right)=\eta_{d}E_{d}\left(r\right)+E_{s}\left(r,\;v^{*}≔1\right),$$

with

(23) $$E_{s}\left(r,v^{*}≔1\right)=\frac{P_{\mathrm{in}}TN_{\mathrm{ss}}b_{0}}{f^{2}}{\left[1+{\left(\frac{r}{fl}\right)}^{2}\right]}^{\frac{s}{2}}.$$

**Figure 16:**
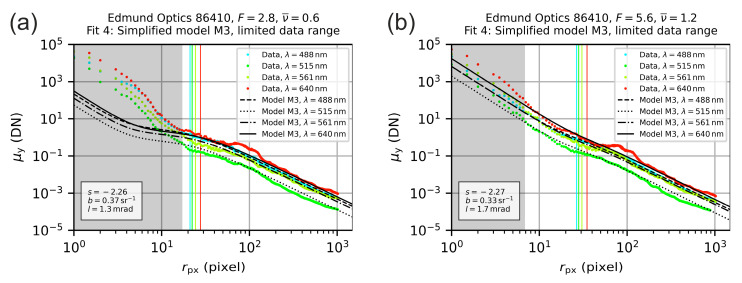
Radial irradiance distribution for the lens Edmund Optics 86410 operated with an f-number of (**a**) F=2.8 and (**b**) F=5.6. The colored points are the measured values derived from the analysis of the camera images. The black lines are the result of fitting the theoretical model M3 to the data within a limited range of the radial coordinate.

The results of a curve fitting with this simplified model M3 are shown in the graphs of Figure 16. The fitting was performed on the previous data using the limited range of the radial coordinate r as described above, see model M2. Although, the resulting fit parameters using model M3 are different to those of model M1, the course of the corresponding fitted curves is similar.

#### 5.3.4. Summary of the Fit Procedure

For each measurement series, we performed the fit procedure as described before:

- Fit 1: Curve fitting with our original model M1 to the full pixel range of the measurement series.
- Fit 2: Curve fitting with the auxiliary model M2 to the full pixel range of the measurement series.
- Fit 3: Curve fitting with our original model M1 to a limited range of data using fit parameter $p_{2}$ gained with fit 2.
- Fit 4: Curve fitting with the simplified model M3 to a limited range of data using fit parameter $p_{2}$ gained with fit 2.

The results of the fitting procedure are presented in detail in Appendix A and are summarized in the following Section 6.

## 6. Results

In this section, we present a summary of the results of the various lens scattering analyses and a subsequent statistical evaluation of the derived scatter parameters. Detailed results of the fitting procedure for each lens can be found tabulated in Appendix A.

**Figure 17:**
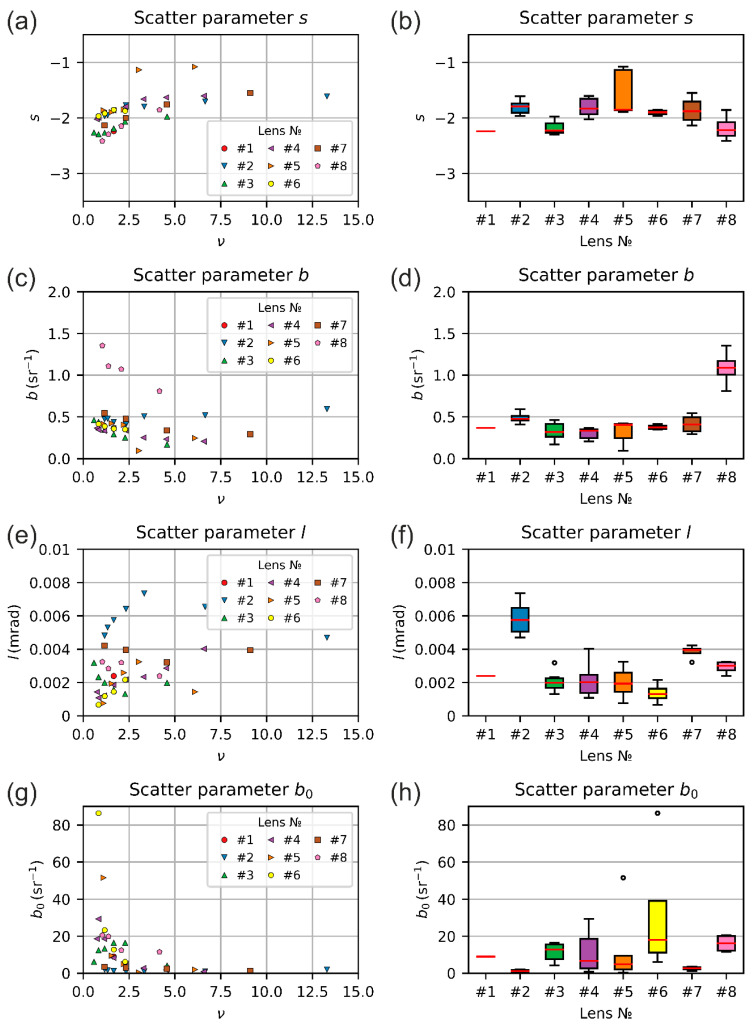
Results of the lens scattering analyses. The numbering of the data corresponds to the lens numbers given in Table 5 or Table 7. (**a**,**c**,**e**,**g**): Scatter parameters s, b, l and $b_{0}$ as a function of the truncation factor ν. (**b**,**d**,**f**,**h**): Box plots of the scatter parameters s, b, l and $b_{0}$ for the different lenses.

Figure 17 shows the results for the scatter parameters. In the left column, Figure 17a,c,e,g shows the values of the scatter parameters s, b, l and $b_{0}$ as a function of the truncation factor ν, respectively. Each data point corresponds to a measurement series, as defined in Section 3. The data sets for the different lenses are distinguished by the color and shape of the data points. The numbering of the legend is equal to the numbering of the lenses as used in Table 5 and Table 7. The scatter parameters s, b and l were obtained directly from Fit 3, whereas scatter parameter $b_{0}$ was calculated using the relation $b_{0}=b{\left(100l\right)}^{s}$; see Equation (9). The scatter parameters are pure material parameters and are independent from the truncation factor ν. However, looking at these four graphs, we can see that the fluctuation of the values seems not always to be of statistical nature. In some cases, for example the scatter parameter l for lens #2 (Edmund Optics 67715), the data points seem to lie on a bended curve. We have no explanation for this observation, but attributed this to the simplicity of our theoretical models. The derivation of the model curve of Equation (1) was accompanied by several assumptions and simplifications to keep the equations manageable. A deeper investigation of this behavior would be of future interest. Furthermore, we can see some outliers in the data, for example for scatter parameter s regarding lens #5 (Navitar NMV-75). We therefore decided to use the median as a robust estimator for the central tendency of the scatter parameters for each lens.

The results of the statistical analysis for the scatter parameters s, b, l and $b_{0}$ are plotted as box plots in Figure 17b,d,f,h, respectively. The results are listed in Table 7: the median, the interquartile range (IQR, difference of third and first quartile) and additionally the quartile coefficient of dispersion (QCD). The QCD is defined as a ratio of IQR to the sum of the first and third quartile and describes the dispersion of the values. From the box plots of Figure 17 we can learn that for the camera lenses (lenses #1–#7), the medians of the scatter parameters s and b do not vary much. For the scatter parameter l, the variation of the median was somewhat stronger, caused mainly by lenses #2 (Edmund Optics 67715) and #7 (Schneider-Kreuznach Xenoplan 2.8/50). Additionally, for the scatter parameter $b_{0}$, there was some larger fluctuation of the median value.

The results of Table 7 build a promising basis to state a generic set of scatter parameters for COTS camera lenses with regard to analyze the incapacitation of sensors when dazzled. In other words, these results also help in terms of laser safety considerations or laser safety calculations as performed in reference [7]. Calculating the median of the scatter parameters of all camera lenses (lens #1–#7, excluding lens #8) results in the following generic set of scatter parameters for the data we measured:

$$\tilde{s}=-1.88;\;\tilde{b}=0.37\;sr^{-1};\;{\tilde{b}}_{0}=6.74\;sr^{-1};\;\tilde{l}=2.02\;\mathrm{mrad}$$

Unfortunately, these values do not fulfill Equation (9): ${\tilde{b}}_{0}=6.74\;sr^{-1}\neq \tilde{b}{\left(100\cdot \tilde{l}\right)}^{\tilde{s}}=7.47$. This relation is only valid for the scatter parameters obtained for each measurement series by the various curve fits (listed in Appendix A). Neither the median values for each data set (corresponding to a camera lens) stated in Table 7 do fulfill the relation nor does the generic set of scatter parameters stated above.

In order to be able to deduce a consistent set of scatter parameters, the values given above have to be adjusted accordingly. For example, one could simply increase scatter parameter $\tilde{s}$ (to a less negative value), decrease scatter parameter $\tilde{b}$, increase ${\tilde{b}}_{0}$ or increase scatter parameter $\tilde{l}$ until the relation of Equation (9) is valid, but this would be interpreted like a purely arbitrary approach. Thus, we chose another way by changing all scatter parameters slightly until the relation was fulfilled. Based on our own thoughts, the adaption of the scattering parameters was performed according to the equation

(24) $$\frac{{\tilde{b}}_{0}}{k}=k\cdot \tilde{b}{\left(100\cdot \frac{\tilde{l}}{\sqrt{k}}\right)}^{\sqrt[{3}]{k}\cdot \tilde{s}},$$

where k is a constant. Equation (24) takes into account that the adaption of the scatter parameters by a constant k may have an influence larger than this factor. For example, a change of scatter parameter $\tilde{l}$ by a factor of 0.98 would result in a change of a factor 0.96 on the right hand side of Equation (9) due to the exponent $\tilde{s}$. This is also true for scatter parameter $\tilde{s}$, which leads to prefactors of $1/\sqrt{k}$ and $\sqrt[{3}]{k}$ for parameters $\tilde{l}$ and $\tilde{s}$, respectively.

Equation (24) is valid for a factor k≈0.974, i.e., all parameters were changed by less than 2.6 percent. By rounding to the first two decimal places, we received a new generic set of scatter parameters, expressed now by the capital letters S, B, $B_{0}$ and L:

$$S=-1.86;\;B=0.36\;{\mathrm{sr}}^{-1};\;B_{0}=6.92;\;L=2.04\;\mathrm{mrad}$$

**Figure 18:**
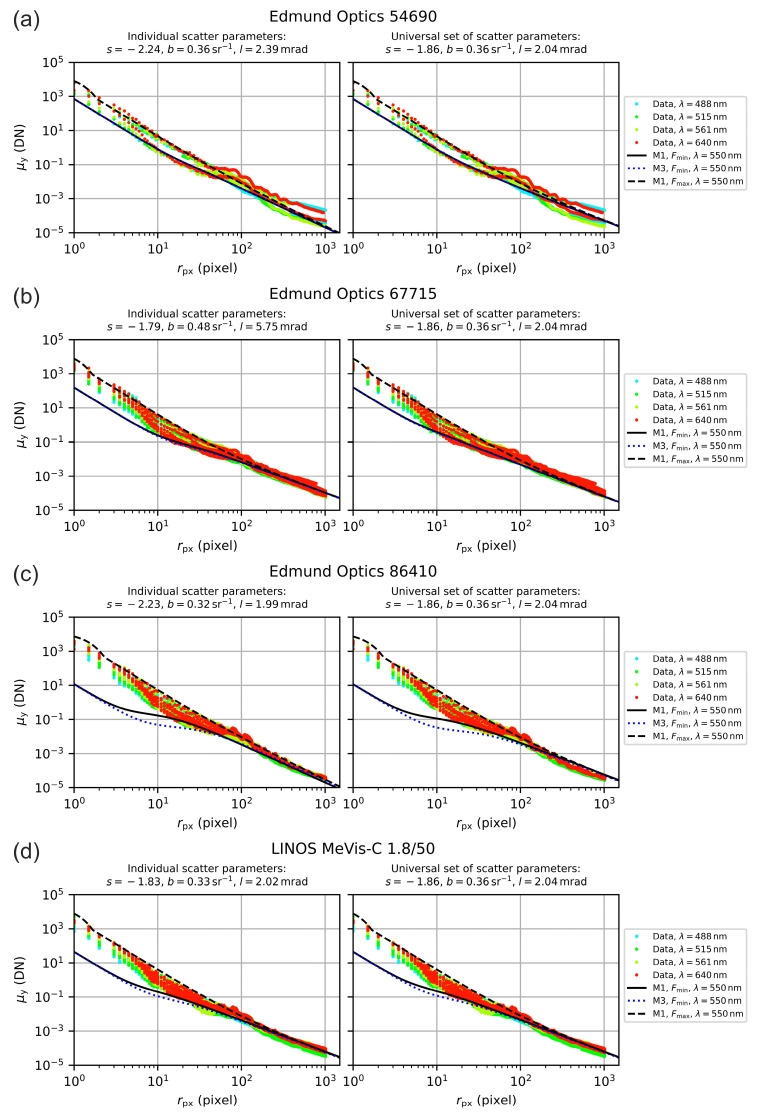
Comparison of measured irradiance profiles and predictions of the theoretical model M1. Calculations were performed both using the individual scatter parameters of the lens (graphs on the left hand side) and the generic set of scatter parameters (graphs on the right hand side). Lenses: (**a**) Edmund Optics 54690, (**b**) Edmund Optics 67715, (**c**) Edmund Optics 86410 and (**d**) LINOS MeVis-C 1.8/50.

**Figure 19:**
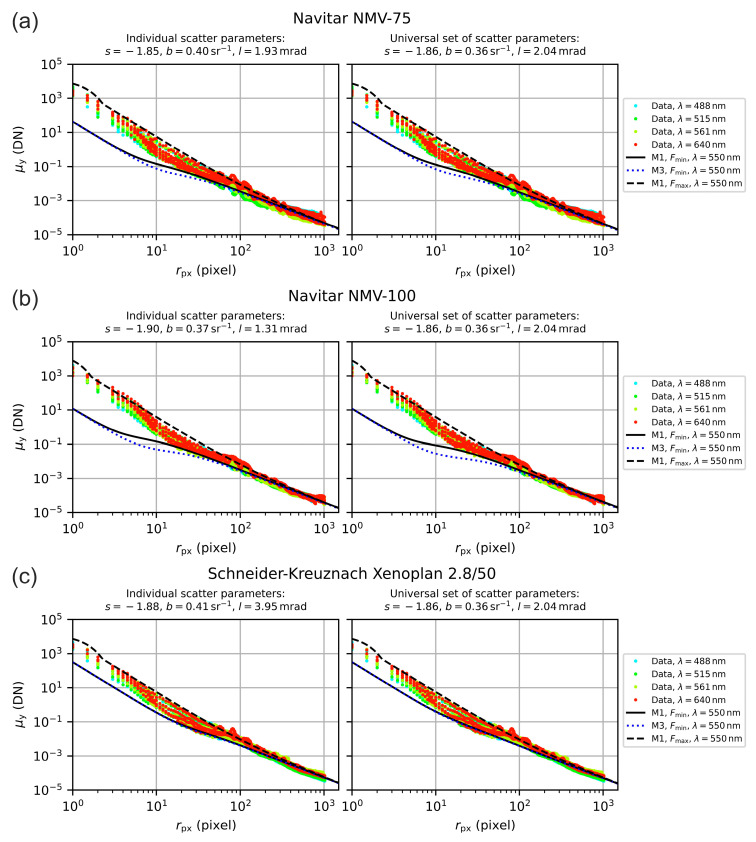
Comparison of measured irradiance profiles and predictions of the theoretical model M1. Calculations were performed both using the individual scatter parameters of the lens (graphs on the left hand side) and the generic set of scatter parameters (graphs on the right hand side). Lenses: (**a**) Navitar NMV-75, (**b**) Navitar NMV-100 and (**c**) Schneider-Kreuznach Xenoplan 2.8/50.

There may be alternative methods to balance the scatter values, but with Figure 18 and Figure 19 we demonstrate the applicability of this method. The figures show the measured irradiance profiles for all seven camera lenses, plotted in separate graphs. Since the incident laser power $P_{\mathrm{in}}$ varied for the different settings of the f-number F, we scaled all the profiles to the same (arbitrarily chosen) input power of 0.1 µW by applying the respective factor of $0.1\;\mu W/P_{\mathrm{in}}$ to the data, in order to normalize the data. The resulting point cloud reflects quite well the value range of the camera signal $\mu_{y}$ as a function of radial coordinate $r_{\mathrm{px}}$.

Furthermore, we plotted theoretical curves regarding the minimum and maximum f-number $F_{\min}$, $F_{\max}$ of each lens. For the theoretical curves, we used the original model M1 (black curves) and additionally the simplified model M3 (blue dotted curve). The curve for model M3 was calculated only for f-number $F_{\min}$, since there was no difference to model M1 in the case of f-number $F_{\mathrm{ma}x}$. All theoretical curves were calculated using the reference wavelength of 550 nm. For the calculations, we used both the individual scatter parameters of the lenses as given by the median values of Table 7 (graphs on the left hand side) and the generic set of scatter parameters S, B and L as stated above (graphs on the right hand side).

We can see that the $F_{\min}$, $F_{\max}$ model curves enclosed quite well the data points for radial coordinates r≳10 pixel=55 μm. It is clear that the curves calculated with the individual scatter parameters of the camera lenses gave more accurate results than the generic set of scatter parameters. However, the generic set seemed to be a good choice if scatter parameters of a camera lens are unknown. Depending on the camera lens, sometimes differences occurred between the result of model M1 and M3. In case of lenses no. 1, 2 and 7 (Edmund Optics 54690 and 67715, Schneider-Kreuznach Xenoplan 2.8/50), there was no difference visible. For lenses no. 3 and 6 (Edmund Optics 86410 and Navitar NMV-100), there appeared larger difference for radial coordinates r≲50 pixel=275 μm.

Regarding the sensor’s incapacitation, this difference of models M1 and M3 may play a role for dazzle scenarios, especially when the laser beam diameter is smaller or similar to size of the lens aperture ($\nu <\sqrt{2}$) and for rather small dazzle spots with $r_{\mathrm{dazzle}}≲50\;\mathrm{pixel}$s. For most practical applications, where the laser source is typically further away and the laser beam overspills the optics diameter, the simplified model M3 should be adequate.

## 7. Simulation of Stray Light Irradiance Using the Optical Engineering Software FRED

Our determination of a generic set of scatter parameters for camera lenses was based on measurements using a sample of seven different COTS camera lenses. It is clear that this set of values will not cover adequately all kinds of camera lenses. In order to compare the results of our theoretical stray light model (using the generic set of scatter parameters S, B, $B_{0}$ and L) with stray light distributions of other typical camera lenses, we utilized the optical engineering software FRED from Photon Engineering. Using this software, we performed stray light analyses for two camera lenses of the double Gauss type, since it is stated that “35-mm SLR normal lenses are invariably Double-Gauss types” [17]. Furthermore, we modeled the achromatic doublet lens (Thorlabs AC254-050-A) also used in our measurements.

### 7.1. Layout of the Stray Light Simulation

**Figure 20:**
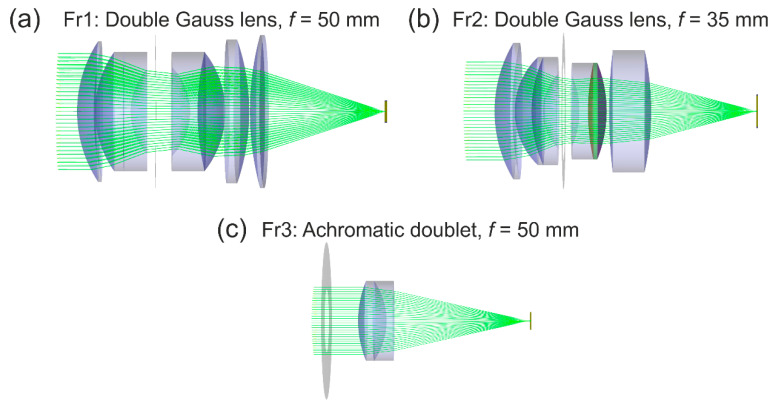
Optical layout of modeled (camera) lenses: (**a**) Lens Fr1 of double Gauss type, (**b**) lens Fr2 of double Gauss type and (**c**) achromatic doublet lens Fr3.

The lenses modeled with FRED are listed in Table 8 and the corresponding optical layouts are shown in Figure 20. The optical layouts for the double Gauss lenses were taken from reference [18]; the optical layout of the achromatic doublet lens was provided by the manufacturer.

For the stray light simulation, we used the settings for the FRED software listed in Appendix C, Table A9. The laser beam was simulated by a source grid of a defined number of input rays, homogeneously distributed within a predefined aperture. The diameter of that aperture was adjusted in that way that the source grid was slightly larger than the maximum input aperture of the considered lens. The total input power of 2 µW was distributed to the rays of each source grid in such a way, to receive always a Gaussian beam with a $d_{86}$-diameter of 21.1 mm. The wavelength of the simulated light source was set to 550 nm. These settings corresponded largely to those of our experimental setup.

The simulation of light scattering comprised of two different kinds of scatter functions. First, scattering of light at the rough surfaces of the optical elements according to Harvey’s scatter model and, second, scattering of light at the housing, at the rim of the apertures and at the lens’ edges. For the second, we assumed Lambertian reflection of 4% reflectivity. As scatter parameters for the Harvey scatter model, we either used our generic set of scatter parameters as described above (lens Fr1/Fr2) or, in the case of the achromatic doublet (lens Fr3), the measured scatter parameters as stated in Table 7 (see results for lens #8). Since we had no exact CAD models of lens barrels for the double Gauss lens systems, their housing was simply simulated by conical tubes connecting the edges of adjacent optical elements. More sophisticated methods for stray light control, like baffles and vanes, special paints and surface treatments were not simulated. In the case of the achromatic doublet lens, a cylindrical tube was simulated as a lens barrel, which is close to reality since the housing for the achromatic doublet lens was built using a standard opto-mechanical tube system; see Figure 7h.

For the analysis of stray light in the focal plane of a sensor, we defined a detector of dimensions 5.5 mm × 5.5 mm with 1000 pixels × 1000 pixels. This results in a pixel size of 5.5 µm, which is identical to the pixel size of the imaging sensor we used in our measurements. The detector was always positioned at the geometric focus of the respective lens.

The basic principle of stray light simulations is that each of the input beams that will be refracted/reflected at the lenses’ surfaces or mechanical parts according to geometric optics, generates a large number of scattered rays. Thus, the number of rays to be simulated in total is a high multiple of each single input ray. Consequently, the number of rays reaching the detector depends not only on the number of input rays, but also on the vast number of parameters of the simulation software, which influences the accuracy of the simulation. That is, not only the number of input rays but also the number of scattered rays have a major impact on the outcome. It can be said, that the larger the number of input rays, the better the results for the central spot that is dominated by diffraction and aberrations. On the other side, the scattered irradiance distribution can be simulated better when using a large number of scatter rays. Therefore, producing realistic results using such an optical engineering software requires both a huge number of input and scatter rays, which requires a huge amount of computational power. Here, we present first the results of our stray light simulation. Since the number of applicable rays was limited by the computer hardware, we decided to privilege the scattering part for this publication. This means that we expect and accept deviations between simulations and measurements in the regime where diffraction dominates the irradiance signal. More details on the simulation process and an extensive analysis of our results will be presented in a dedicated publication.

### 7.2. Simulation Results

**Figure 21:**
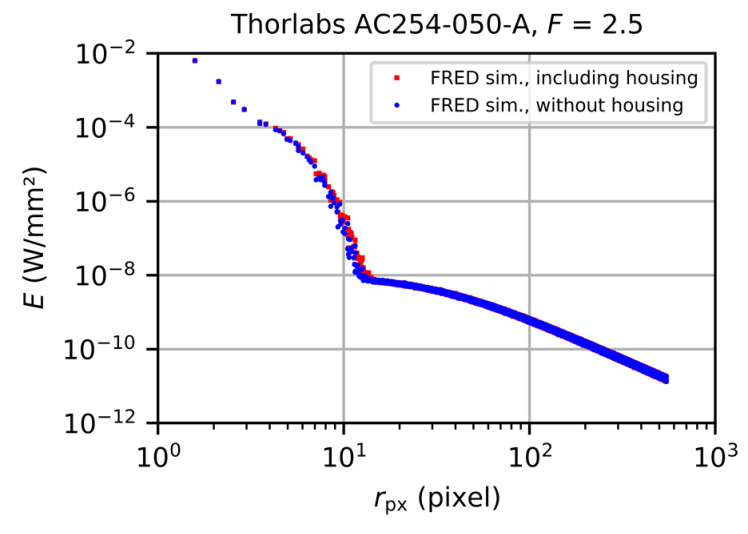
Simulated focal plane irradiance for the achromatic doublet lens. Red data points: simulation including housing. Blue data points: simulation without housing.

In the course of our investigations, we observed that the simulations performed with or without lens housing nearly show the same results, as depicted in Figure 21 for the case of the achromatic doublet lens. The simulated irradiance data are plotted as a function of the radial distance with respect to the center of the laser spot for the two cases with lens housing (red data points) and without lens housing (blue data points). Both curves had more or less the same course, especially for larger values of the radial coordinate, where scattered light from the lenses itself dominated the signals in the focal plane.

This result can be attributed to the specific cases (paraxial setup) we investigated and we simulated here: light only impinges on the camera lens along the optical axis without hitting directly the housing. In turn this means that only scattered light from the optical elements will reach the housing and subsequently the sensor. In future, the effects of the oblique incidence of light shall be examined, where the housing is directly hit by the impinging light.

**Figure 22:**
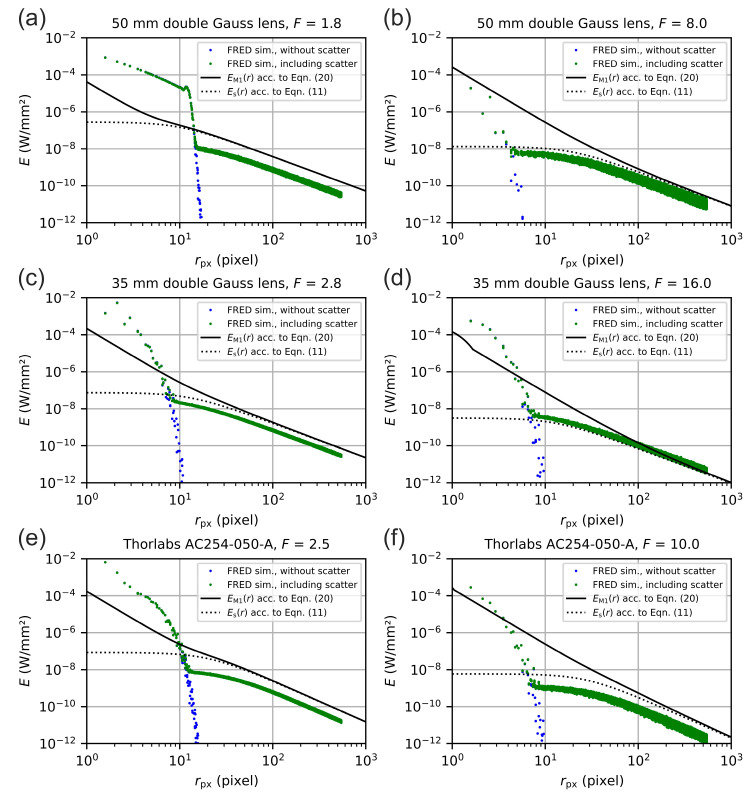
Modeling of the focal plane irradiance as a function of radial distance to the center of the laser spot for the following lens/f-number combinations: 50 mm double Gauss lens with F=1.8 (**a**) and F=8.0 (**b**); 35 mm double Gauss lens with F=2.8 (**c**) and F=16.0 (**d**) and achromatic doublet lens: F=2.5 (**e**) and F=10.0 (**f**).

In Figure 22, we plotted the simulated irradiance data as a function of radial distance with respect to the center of the laser spot (colored data points) for the three modeled lenses. For comparison, the graphs show results for raytracing without any scattering (blue data points) and with lens scattering (green data points). All these data was simulated without housing since a full scattering investigation would be highly time consuming, and would not lead to a better understanding of details. Furthermore, we also plotted the result of our theoretical model M1 using the same parameters as we used for the FRED simulations. The black solid curve shows the irradiance $E_{M1}\left(r\right)$ of model M1 according to Equation (20), which comprises diffraction and scattering of light. The black dashed curve solely shows the scatter part $E_{s}\left(r\right)$ according to Equation (11). The graphs on the left-hand side were results for the smallest f-numbers modeled; the graphs on the right-hand side show results for the largest f-numbers modeled.

For small values of the radial coordinate r, there was a larger difference between the simulated irradiance and the output of our theoretical model. This is unsurprisingly, since the simulation also comprised aberrations whereas our theoretical model did not. Furthermore, as stated above, we adjusted the simulation in that way that the results should be more exact for the scattered part of the simulated irradiance distribution.

For larger values of the radial coordinate, the simulated irradiance values were typically slightly below the values of the theoretical model. The result for the larger f-number of the 35 mm double Gauss lens in Figure 22d is an exception. In this case, the f-number was larger (F=16) compared to the other lenses; the simulated irradiance values were slightly above the model curve. We do not exactly know where these deviations resulted from. This will be investigated in more detail in future work.

However, as a first result, the simulation is in good agreement with the theoretical model. Thus, we could conclude that the theoretical model applied with the generic set of scatter parameters allows for an appropriate estimate of the irradiance distribution of typical camera lenses.

## 8. Summary

In this publication, we present our measurements to assess the scattering parameters of commercial off-the-shelf (COTS) camera lenses. For this, the spatial irradiance distribution of laser light at the focal plane of different camera lenses was measured using a camera as a detector. Assuming rotational symmetry of the irradiance distribution, the image data was used to derive radial irradiance profiles. Subsequently, a simple theoretical model for irradiance distribution calculations was used to perform curve fitting to the experimentally measured irradiance profiles in order to extract the scatter parameters of the camera lenses. These scatter parameters are related to the well-known 3-parameter Harvey scatter model describing light scattering from the rough surfaces of optical elements. The main outcome of our work shows that the values of the scatter parameters for quite specific types of camera lenses were very similar. This allowed us to state a generic set of scatter parameters for typical COTS camera lenses and, moreover, now will allow us to perform laser safety calculations for sensors even in the case that exact values for the scatter parameters of a camera lens are not available, which was the main motivation for this work.

To our best knowledge, it is the first time that scatter parameters for standard COTS camera lenses were published. However, the model used to extract the scatter parameters was not a rigorous theoretical model of the irradiance distribution within camera lenses. The model was specifically developed to perform laser safety calculations for imaging sensors. It comprised several simplifications and, thus, the scatter parameters presented here are specific and might not be readily applicable for other types of optics. Therefore, we used the FRED optical engineering software to examine whether the stated general set of scatter parameters can also be applied to other camera lenses than those used for the measurements presented here. As a result, we see that in combination with the dedicated theoretical model, the stated general set of scatter parameters allows a good estimation of the focal plane irradiance distribution of camera lenses.

Future work on that topic could comprise the increase of the statistical database by both including further camera lenses and performing more measurements on the currently used camera lenses. Regarding further camera lenses, additional investigations could include, for example, zoom lenses, where the scattering parameters could be measured at different settings of their focal length, or a telephoto lens with a very large focal length (500 mm). For the camera lenses already in use, further research could comprise measurements for different truncation factors by changing the laser beam diameter instead of the f-number. It would also be of interest to test how non-coherent radiation (e.g., by using light of a narrowband LED) would change the stray light distribution. An important point for future work is the validation of laser safety calculations for imaging sensors using the here stated generic set of scatter parameters. The validation could be performed using data acquired with different sensors and different camera lenses, in particular data gathered in free-field conditions. Furthermore, it is of particular interest whether the simplified model already meets the requirements for the laser safety calculations. This would be another step to a further simplification of our model.

## Figures and Tables

**Table 1:** Parameters used for the theoretical model.

Symbol	Unit	Quantity
Laser		
$P_{\mathrm{laser}}$	W	Power
$d_{63}$	m	Beam diameter (1/e)
$d_{86}=\sqrt{2}d_{63}$	m	Beam diameter (1/e^2^)
λ	m	Wavelength
Camera lens		
f	m	Focal length
$d_{\mathrm{ap}}$	m	Aperture/entrance pupil diameter
$F=f/d_{\mathrm{ap}}$		f-number
$N_{\mathrm{oe}}$		Number of optical elements
$N_{\mathrm{ss}}=2\cdot N_{\mathrm{oe}}$		Number of scattering surfaces
T		Transmittance
$s/b/b_{0}/l$	-/sr^−1^/sr^−1^/rad	Scatter parameters
Imaging sensor		
p	m	Pixel size
A	m^2^	Pixel area
η		Total quantum efficiency
$t_{\exp}$	s	Exposure time
$\mu_{y}$	DN (digital number)	Digital signal (gray value)
$\mu_{y.\mathrm{sat}}$	DN	Saturation gray value
$\mu_{y.\min}$	DN	Absolute sensitivity threshold
K	DN/e^−^	Overall system gain
bd	bit	Bit depth
Miscellaneous		
$r/r_{\mathrm{px}}$	m/pixel	Radial coordinate
$\nu =d_{86}/d_{ap}$		Truncation factor
$P_{\mathrm{in}}=P_{\mathrm{laser}}\left(1-\exp\left(-2/\nu^{2}\right)\right)\;$	W	Laser power entering the camera lens
$d_{\mathrm{spot}}=k\lambda F$	m	Laser spot size in the focal plane
k		Spot size constant; see reference [12]

**Table 2:** Parameters of the camera Allied Vision Mako G-419B NIR.

**Specifications by the Manufacturer**	
Resolution	2048 pixels × 2048 pixels
Sensor	CMOSIS/ams CMV4000 NIR
Pixel size p	5.5 µm
Bit depth bd	12
Quantum efficiency η (at 529 nm)	0.79
Saturation capacity $\mu_{e.\mathrm{sat}}$	9500 e^−^
Absolute sensitivity threshold $\mu_{e.\min}$	14.1 e^−^
**Own measurements**	
Overall system gain K	0.399 DN/e^−^
Responsivity R	0.306 DN/photon
Quantum efficiency η (at 635 nm)	0.77
Saturation capacity $\mu_{e.\mathrm{sat}}$	9570 e^−^
Saturation gray value $\mu_{y.\mathrm{sat}}$	3861 DN

**Table 3:** Definition of terms for different measurement processes. CL: camera lens.

Term	Meaning	Output after Data Analysis/Statistical Evaluation	Parameter Changed				
			CL	F	λ	$t_{exp}$	$P_{laser}$
Image acquisition	Recording of a camera image	Radial irradiance profile within a circumscribed area of the imaging sensor	-	-	-	-	-
Measurement	Set of 8 image acquisitions gained using 8 different combinations of exposure time and laser power	Radial irradiance profile for the (almost) complete area of the imaging sensor	-	-	-	×	×
Measurement series	A series of measurements obtained using different laser wavelengths	Wavelength-dependent radial irradiance profiles used for fitting theoretical models to estimate the scatter parameters	-	-	×	×	×
Data set	A set of measurement series for different values of the f-number	A single set of scatter parameters for a specific camera lens	-	×	×	×	×
Data ensemble	The compilation of data sets of all camera lenses	A generic set of scatter parameters valid for all camera lenses	×	×	×	×	×

**Table 4:** Combinations of exposure time $t_{\exp}$ and nominal optical density $OD_{A2}$ of attenuator A2.

Setting №	1	2	3	4	5	6	7	8
$t_{exp}$ **(µs)**	100	100	100	100	100	1000	10^4^	10^5^
**$OD_{A2}$**	4	3	2	1	0	0	0	0

**Table 5:** Lenses used for the investigations. CL: camera lens, AD: achromatic doublet.

Lens №	Model	Type	Focal Length (mm)	Minimum f-Number	No. of Elements	Coating	Price (typ.)
1	Edmund Optics 54690	CL	50	4.0	6	MgF_2_	500 €
2	Edmund Optics 67715	CL	25	1.4	7	BBAR	500 €
3	Edmund Optics 86410	CL	100	2.8	7	BBAR	500 €
4	LINOS MeVis-C 1.8/50	CL	50.6	1.8	7	N/A	700 €
5	Navitar NMV-75	CL	75	2.5	5	N/A	185 €
6	Navitar NMV-100	CL	100	2.8	5	N/A	170 €
7	Schneider-Kreuznach Xenoplan 2.8/50	CL	50.2	2.8	6	N/A	630 €
8	Thorlabs AC254-050-A	AD	50	2.5	2	BBAR	70 €

**Table 6:** f-number settings used for the measurement series and their corresponding mean truncation factor.

Lens	f-Number F/Mean Truncation Factor $\bar{\nu }$							
Edmund Optics 54690	4.0/1.7	18.0/7.5						
Edmund Optics 67715	1.4/1.2	1.6/1.3	2.0/1.7	2.8/2.3	4.0/3.3	8.0/6.6	16.0/13.3	
Edmund Optics 86410	2.8/0.6	4.0/0.8	5.6/1.2	8.0/1.7	11.0/2.3	22.0/4.6		
LINOS MeVis-C 1.8/50	1.8/0.7	2.0/0.8	2.8/1.1	4.0/1.6	5.6/2.3	8.0/3.3	11.0/4.5	16.0/6.6
Navitar NMV-75	2.5/0.7	4.0/1.1	5.6/1.6	8.0/2.2	11.0/3.0	22.0/6.1		
Navitar NMV-100	2.8/0.6	4.0/0.8	5.6/1.2	8.0/1.7	11.0/2.3	16.0/3.3		
Schneider-Kreuznach Xenoplan 2.8/50	2.8/1.2	5.6/2.3	11.0/4.6	22.0/9.1				
Thorlabs AC254-050-A	2.5/1.0	3.3/1.4	5.0/2.1	10.0/4.2				

**Table 7:** Results of the statistical analysis for the scatter parameters s, b, l and $b_{0}$. IQR: interquartile range, QCD: quartile coefficient of dispersion.

Lens №	Model		s	$b\;\left(sr^{-1}\right)$	$b_{0}$	l (mrad)
1	Edmund Optics 54690	Median	−2.24	0.36	8.96	2.39
		IQR	0.00	0.00	0.00	0.00
		QCD	−0.00	0.00	0.00	0.00
2	Edmund Optics 67715	Median	−1.79	0.48	1.23	5.75
		IQR	0.17	0.06	0.83	1.42
		QCD	−0.05	0.06	0.30	0.12
3	Edmund Optics 86410	Median	−2.23	0.32	12.85	1.99
		IQR	0.17	0.15	7.92	0.56
		QCD	−0.04	0.23	0.34	0.14
4	LINOS MeVis-C 1.8-50	Median	−1.83	0.33	6.74	2.02e
		IQR	0.28	0.10	15.99	1.09
		QCD	−0.08	0.17	0.76	0.28
5	Navitar NMV-75	Median	−1.85	0.40	4.93	1.93
		IQR	0.73	0.17	7.52	1.15
		QCD	−0.24	0.26	0.65	0.29
6	Navitar NMV-100	Median	−1.90	0.37	18.01	1.31
		IQR	0.06	0.04	27.92	0.58
		QCD	−0.02	0.05	0.56	0.22
7	Schneider Kreuznach Xenoplan 2.8/50	Median	−1.88	0.41	2.76	3.95
		IQR	0.33	0.17	0.98	0.27
		QCD	−0.09	0.21	0.18	0.03
8	Thorlabs AC254-050-A	Median	−2.22	1.09	16.14	3.02
		IQR	0.25	0.17	7.87	0.48
		QCD	−0.06	0.08	0.24	0.08

**Table 8:** Camera lenses modeled using the optical engineering software FRED.

Lens №	Type	Focal Length (mm)	f-Numbers F	No. of Elements
Fr1	Double Gauss	50	1.8/2.0/2.8/4.0/5.6/8.0	7
Fr2	Double Gauss	35	2.8/4.0/5.6/8.0/10.0/16.0	6
Fr3	Achromatic Doublet (Thorlabs AC254-050-A)	50.2	2.5/3.3/5.0/10.0	2

**Figure A1:**
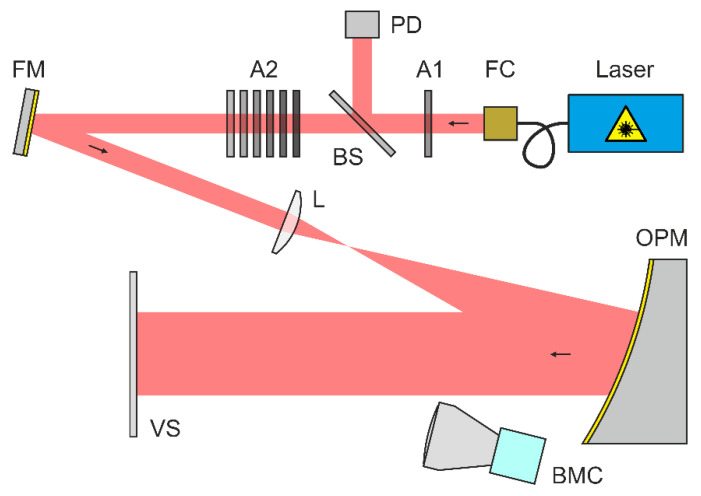
Experimental setup for measuring the laser beam diameter.

**Figure A2:**
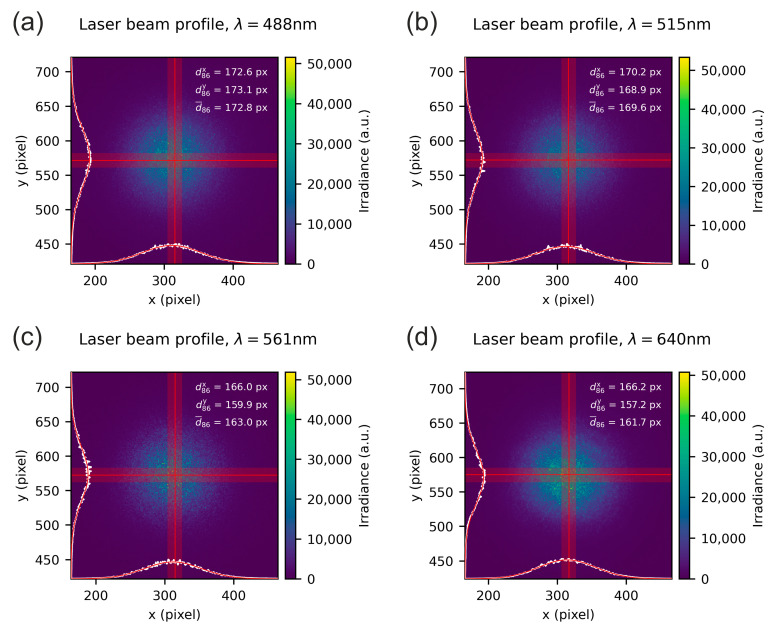
Irradiance distribution of the laser beam at the viewing screen for laser wavelengths of (**a**) 488 nm, (**b**) 515 nm, (**c**) 561 nm and (**d**) 640 nm. Additionally, vertical and horizontal profile lines through the centroid of the irradiance distribution are shown.

**Figure A3:**
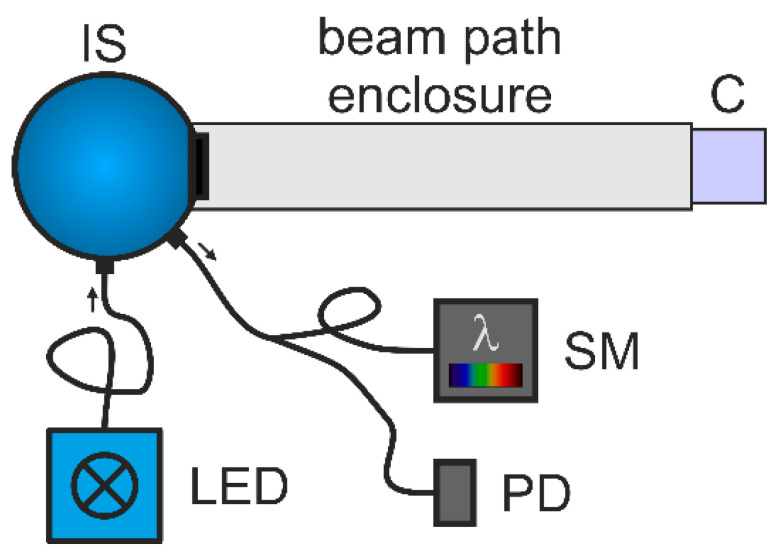
Schematic drawing of the experimental setup to characterize the camera according to the EMVA 1288 standard. LED: light-emitting diode, IS: integrating sphere, PD: reference photodiode, SM: spectrometer, C: camera.

**Figure A4:**
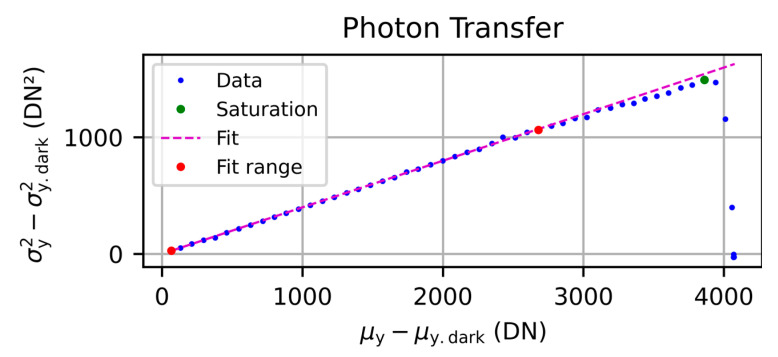
Photon transfer curve for the camera Allied Vision Mako G-419B NIR.

**Figure A5:**
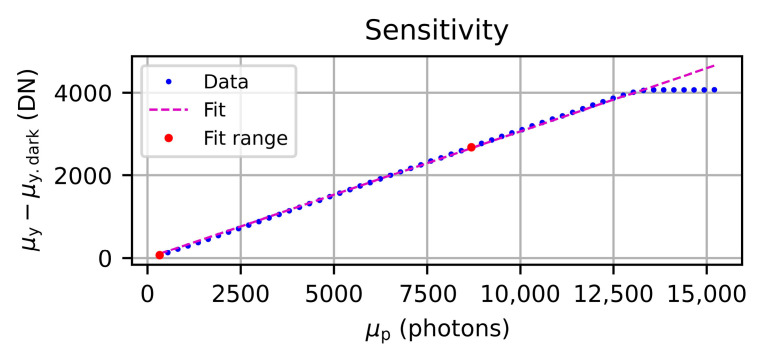
Sensitivity curve for the camera Allied Vision Mako G-419B NIR.

## Appendix A. Results of the Data Analysis

As a result of our data exploitation, Table A1, Table A2, Table A3, Table A4, Table A5, Table A6, Table A7 and Table A8 present the scatter parameters s, b and l, which served as curve fitting parameters to fit our data by means of equations derived from our models M1–M3. Additionally, the scatter parameter $b_{0}$, calculated from Equation (9), is presented. The fit procedure, explained in detail in Section 5.3, was based on four individual curve fittings. For the convenience of the reader, the various curve fits are repeated here:

- Fit 1: Curve fitting with our original model M1 to the full pixel range of the measurement series.
- Fit 2: Curve fitting with the auxiliary model M2 to the full pixel range of the measurement series.
- Fit 3: Curve fitting with our original model M1 to a limited range of data using fit parameter $p_{2}$ gained with fit 2.
- Fit 4: Curve fitting with the simplified model M3 to a limited range of data using fit parameter $p_{2}$ gained with fit 2.

The measurements were performed on eight different lenses and for various settings of f-number F/mean truncation factor $\bar{\nu }$. All results were converted to the reference wavelength of 550 nm. Each table also contains the median of the various scatter parameters. The values of fit no. 3 are shown in bold letters, since these values were used for the statistical analysis of Section 6. To calculate the median values for a specific lens, usually all the fit results for this lens were taken into account. However, in some cases the fit value for the scatter parameter l reached its lower limit of 0.5 mrad. These cases are highlighted by the red letters in the tables. When this occurred for fit no. 3, the results for this measurement series were excluded from the calculation of the median values, since these fit results were assumed to describe the data not properly. The excluded data is highlighted by the orange background in the tables.

**Table A1:** Fit results for the lens Edmund Optics 54690.

Edmund Optics 54690							
λ (nm)	F	$\bar{\nu }$	Fit №	s	$b\;\left(sr^{-1}\right)$	l (mrad)	$b_{0}\;\left(sr^{-1}\right)$
550	4.0	1.7	1	−2.24	0.36	2.37	9.07
			2	−2.24	0.36	2.40	8.86
			3	−2.24	0.36	2.39	8.96
			4	−2.24	0.36	2.39	8.96
550	18.0	7.5	1	−1.56	0.50	0.50	53.79
			2	−1.56	0.50	0.50	53.79
			3	−1.56	0.50	0.50	53.79
			4	−1.56	0.50	0.50	53.79
		Median	1	−2.24	0.36	2.37	9.07
			2	−2.24	0.36	2.40	8.86
			3	**−2.24**	**0.36**	**2.39**	**8.96**
			4	−2.24	0.36	2.39	8.96

**Table A2:** Fit results for the lens Edmund Optics 67715.

Edmund Optics 67715							
λ (nm)	F	$\bar{\nu }$	Fit №	s	$b\;\left(sr^{-1}\right)$	l (mrad)	$b_{0}\;\left(sr^{-1}\right)$
550	1.4	1.2	1	−2.00	0.50	0.50	200.60
			2	−1.99	0.50	6.39	1.22
			3	−1.97	0.47	4.81	1.99
			4	−1.97	0.48	4.03	2.89
550	1.6	1.3	1	−1.91	0.42	0.50	127.59
			2	−1.96	0.49	5.92	1.37
			3	−1.95	0.48	5.31	1.64
			4	−1.96	0.49	5.05	1.85
550	2.0	1.7	1	−1.86	0.43	5.58	1.29
			2	−1.87	0.44	5.86	1.19
			3	−1.87	0.44	5.75	1.23
			4	−1.87	0.44	5.75	1.23
550	2.8	2.3	1	−1.77	0.41	6.41	0.89
			2	−1.77	0.41	6.44	0.89
			3	−1.77	0.41	6.41	0.89
			4	−1.77	0.41	6.41	0.89
550	4.0	3.3	1	−1.80	0.51	7.37	0.88
			2	−1.80	0.51	7.36	0.88
			3	−1.80	0.51	7.36	0.88
			4	−1.80	0.51	7.36	0.88
550	8.0	6.6	1	−1.70	0.52	6.55	1.06
			2	−1.70	0.52	6.54	1.07
			3	−1.70	0.52	6.54	1.06
			4	−1.70	0.52	6.54	1.06
550	16.0	13.3	1	−1.61	0.59	4.70	2.00
			2	−1.61	0.59	4.70	2.00
			3	−1.61	0.59	4.70	2.00
			4	−1.61	0.59	4.70	2.00
		Median	1	−1.80	0.50	5.58	1.29
			2	−1.80	0.50	6.39	1.19
			3	**−1.80**	**0.48**	**5.75**	**1.23**
			4	−1.80	0.49	5.75	1.23

**Table A3:** Fit results for the lens Edmund Optics 86410.

Edmund Optics 86410							
λ (nm)	F	$\bar{\nu }$	Fit №	s	$b\;\left(sr^{-1}\right)$	l (mrad)	$b_{0}\;\left(sr^{-1}\right)$
550	2.8	0.6	1	−2.31	0.48	0.50	481.75
			2	−2.27	0.47	3.46	5.21
			3	−2.26	0.46	3.18	6.12
			4	−2.26	0.37	1.31	36.47
550	4.0	0.8	1	−2.20	0.38	0.50	275.46
			2	−2.31	0.45	2.53	10.57
			3	−2.30	0.44	2.33	12.42
			4	−2.30	0.38	1.37	36.39
550	5.6	1.2	1	−2.27	0.34	1.98	13.44
			2	−2.27	0.34	2.00	13.14
			3	−2.27	0.34	1.99	13.28
			4	−2.27	0.33	1.65	19.60
550	8.0	1.7	1	−2.19	0.29	1.58	16.35
			2	−2.19	0.29	1.58	16.37
			3	−2.19	0.29	1.58	16.39
			4	−2.19	0.29	1.58	16.39
550	11.0	2.3	1	−2.06	0.25	1.32	16.41
			2	−2.07	0.25	1.43	14.17
			3	−2.06	0.25	1.32	16.41
			4	−2.06	0.25	1.32	16.41
550	22.0	4.6	1	−1.98	0.17	1.98	4.13
			2	−1.98	0.17	1.98	4.13
			3	−1.98	0.17	1.98	4.13
			4	−1.98	0.17	1.98	4.13
		Median	1	−2.20	0.32	1.45	16.38
			2	−2.23	0.32	1.99	11.85
			3	**−2.23**	**0.32**	**1.99**	**12.85**
			4	−2.23	0.31	1.48	18.00

**Table A4:** Fit results for the lens LINOS MeVis-C 1.8/50.

LINOS MeVis-C 1.8/50							
λ (nm)	F	$\bar{\nu }$	Fit №	s	$b\;\left(sr^{-1}\right)$	l (mrad)	$b_{0}\;\left(sr^{-1}\right)$
550	1.8	0.7	1	−2.09	0.42	0.50	220.65
			2	−2.04	0.38	2.53	6.31
			3	−2.02	0.36	1.43	18.56
			4	−2.03	0.36	0.79	62.88
550	2.0	0.8	1	−2.05	0.38	0.50	174.92
			2	−2.01	0.36	1.99	9.14
			3	−2.00	0.34	1.08	29.33
			4	−2.00	0.35	0.68	77.41
550	2.8	1.1	1	−1.92	0.33	0.50	102.63
			2	−1.92	0.33	1.50	12.81
			3	−1.92	0.33	1.22	18.52
			4	−1.93	0.34	1.09	24.68
550	4.0	1.6	1	−1.86	0.36	1.77	9.06
			2	−1.86	0.36	1.90	7.97
			3	−1.86	0.36	1.86	8.28
			4	−1.86	0.36	1.86	8.28
550	5.6	2.3	1	−1.80	0.34	2.17	5.20
			2	−1.80	0.34	2.18	5.18
			3	−1.80	0.34	2.18	5.20
			4	−1.80	0.34	2.18	5.20
550	8.0	3.3	1	−1.66	0.25	2.34	2.79
			2	−1.66	0.25	2.34	2.79
			3	−1.66	0.25	2.34	2.79
			4	−1.66	0.25	2.34	2.79
550	11.0	4.5	1	−1.63	0.23	2.86	1.77
			2	−1.63	0.23	3.13	1.56
			3	−1.63	0.23	2.86	1.77
			4	−1.63	0.23	2.86	1.77
550	16.0	6.6	1	−1.60	0.21	4.02	0.89
			2	−1.60	0.21	4.02	0.89
			3	−1.60	0.21	4.02	0.89
			4	−1.60	0.21	4.02	0.89
		Median	1	−1.83	0.33	1.97	7.13
			2	−1.83	0.33	2.26	5.74
			3	**−1.83**	**0.33**	**2.02**	**6.74**
			4	−1.83	0.34	2.02	6.74

**Table A5:** Fit results for the lens Navitar NMV-75.

Navitar NMV-75							
λ (nm)	F	$\bar{\nu }$	Fit №	s	$b\;\left(sr^{-1}\right)$	l (mrad)	$b_{0}\;\left(sr^{-1}\right)$
550	2.5	0.7	1	−2.01	0.56	0.50	228.89
			2	−1.92	0.47	0.50	145.74
			3	−1.93	0.48	0.50	155.39
			4	−1.95	0.52	0.50	178.72
550	4.0	1.1	1	−1.86	0.41	0.50	109.53
			2	−1.87	0.42	0.97	32.66
			3	−1.86	0.42	0.75	51.49
			4	−1.87	0.43	0.61	80.39
550	5.6	1.6	1	−1.90	0.42	1.93	9.47
			2	−1.90	0.42	1.93	9.48
			3	−1.90	0.42	1.93	9.50
			4	−1.90	0.42	1.93	9.50
550	8.0	2.2	1	−1.85	0.40	2.58	4.93
			2	−1.85	0.40	2.58	4.93
			3	−1.85	0.40	2.58	4.93
			4	−1.85	0.40	2.58	4.93
550	11.0	3.0	1	−1.13	0.10	3.25	0.34
			2	−1.13	0.10	3.25	0.34
			3	−1.13	0.10	3.25	0.34
			4	−1.13	0.10	3.25	0.34
550	22.0	6.1	1	−1.08	0.25	1.44	1.98
			2	−1.08	0.25	1.44	1.98
			3	−1.08	0.25	1.44	1.98
			4	−1.08	0.25	1.44	1.98
		Median	1	−1.85	0.40	1.93	4.93
			2	−1.85	0.40	1.93	4.93
			3	**−1.85**	**0.40**	**1.93**	**4.93**
			4	−1.85	0.40	1.93	4.93

**Table A6:** Fit results for the lens Navitar NMV-100.

Navitar NMV−100							
λ (nm)	F	$\bar{\nu }$	Fit №	s	$b\;\left(sr^{-1}\right)$	l (mrad)	$b_{0}\;\left(sr^{-1}\right)$
550	2.8	0.6	1	−2.21	0.67	0.50	506.52
			2	−1.98	0.46	0.50	172.45
			3	−1.99	0.46	0.50	175.15
			4	−2.00	0.47	0.50	189.53
550	4.0	0.8	1	−1.99	0.43	0.50	164.88
			2	−1.98	0.42	1.09	33.84
			3	−1.97	0.41	0.66	86.34
			4	−1.98	0.43	0.50	159.53
550	5.6	1.2	1	−1.92	0.38	1.16	23.96
			2	−1.92	0.39	1.20	22.70
			3	−1.92	0.39	1.18	23.26
			4	−1.92	0.39	0.97	34.56
550	8.0	1.7	1	−1.85	0.36	1.45	12.76
			2	−1.85	0.36	1.45	12.77
			3	−1.85	0.36	1.45	12.77
			4	−1.85	0.36	1.45	12.77
550	11.0	2.3	1	−1.88	0.35	2.16	6.13
			2	−1.88	0.35	2.26	5.71
			3	−1.88	0.35	2.16	6.13
			4	−1.88	0.35	2.16	6.13
550	16.0	3.3	1	-0.75	0.07	0.50	0.65
			2	-0.75	0.07	0.51	0.64
			3	-0.75	0.07	0.50	0.65
			4	-0.75	0.07	0.50	0.65
		Median	1	−1.90	0.37	1.30	18.36
			2	−1.90	0.37	1.32	17.74
			3	**−1.90**	**0.37**	**1.31**	**18.01**
			4	−1.90	0.37	1.21	23.66

**Table A7:** Fit results for the lens Schneider Kreuznach Xenoplan 2.8/50.

Schneider Kreuznach Xenoplan 2.8/50							
λ (nm)	F	$\bar{\nu }$	Fit №	s	$b\;\left(sr^{-1}\right)$	l (mrad)	$b_{0}\;\left(sr^{-1}\right)$
550	2.8	1.2	1	−2.07	0.47	0.50	228.81
			2	−2.15	0.56	4.62	2.92
			3	−2.14	0.54	4.22	3.43
			4	−2.15	0.54	3.53	5.05
550	5.6	2.3	1	−2.00	0.48	3.97	3.03
			2	−2.00	0.48	3.96	3.04
			3	−2.00	0.48	3.96	3.04
			4	−2.00	0.48	3.96	3.04
550	11.0	4.6	1	−1.76	0.34	3.22	2.47
			2	−1.76	0.34	3.28	2.40
			3	−1.76	0.34	3.22	2.47
			4	−1.76	0.34	3.22	2.47
550	22.0	9.1	1	−1.55	0.29	3.94	1.23
			2	−1.55	0.29	3.94	1.23
			3	−1.55	0.29	3.94	1.23
			4	−1.55	0.29	3.94	1.23
		Median	1	−1.88	0.40	3.58	2.75
			2	−1.88	0.41	3.95	2.66
			3	**−1.88**	**0.41**	**3.95**	**2.76**
			4	−1.88	0.41	3.74	2.76

**Table A8:** Fit results for the lens Thorlabs AC254-050-A.

Thorlabs AC254-050-A							
λ (nm)	F	$\bar{\nu }$	Fit №	s	$b\;\left(sr^{-1}\right)$	l (mrad)	$b_{0}\;\left(sr^{-1}\right)$
550	2.5	1.0	1	−2.48	1.48	0.50	2484.40
			2	−2.44	1.41	3.92	13.82
			3	−2.42	1.36	3.24	20.56
			4	−2.43	1.21	2.43	37.79
550	3.3	1.4	1	−2.24	1.00	0.50	813.58
			2	−2.30	1.12	3.02	17.66
			3	−2.29	1.11	2.84	19.88
			4	−2.31	1.12	2.83	20.80
550	5.0	2.1	1	−2.15	1.07	3.19	12.42
			2	−2.15	1.07	3.20	12.37
			3	−2.15	1.07	3.19	12.41
			4	−2.15	1.07	3.19	12.41
550	10.0	4.2	1	−1.86	0.81	2.40	11.48
			2	−1.86	0.81	2.40	11.48
			3	−1.86	0.81	2.39	11.49
			4	−1.86	0.81	2.39	11.49
		Median	1	−2.19	1.03	1.45	413.00
			2	−2.22	1.10	3.11	13.10
			3	**−2.22**	**1.09**	**3.02**	**16.14**
			4	−2.23	1.10	2.63	16.60

## Appendix B

### Appendix B.1. Measurement of the Laser Beam Diameter

The determination of the laser beam diameter at the entrance aperture of the camera lenses was an important task that also required great care. Since commercially available beam profilers are limited to smaller laser beam sizes, we measured the beam diameter using the setup depicted in Figure A1. The setup was largely equal to the setup of Figure 4, except that the camera C and camera lens CL were replaced by a white, diffuse viewing screen VS (Thorlabs EDU-VS1/M). Furthermore, a beam-monitoring camera BMC (camera Allied Vision Mako G-223B NIR + camera lens Edmund Optics 67716) was placed near the off-axis parabolic mirror OPM to observe the viewing screen.

Before the measurement of the laser beam diameter, we ensured that the BMC was focused to the viewing screen. Furthermore, we estimated the magnification $M_{\mathrm{bmc}}$ for the setup by placing a resolution test chart at the position of the viewing screen. Using the known size of the test chart’s features and the corresponding size in the camera image, the magnification was calibrated to be $M_{\mathrm{bmc}}=124.6\;\mu m/\mathrm{pixel}$.

We captured camera images of the laser beam for all four laser wavelengths and dark frames, i.e., without laser illumination. In each case, 10 camera images were acquired and averaged before further analysis. Figure A2 shows the mean images of the laser beam profiles for all four wavelengths. For each image, the centroid of the irradiance distribution was estimated and then a vertical and horizontal profile at the centroid’s position was extracted. For each vertical/horizontal profile, 21 pixel columns/rows were used and averaged. These areas are marked by the red bars in the images of Figure A2. The corresponding profile lines are plotted in white color into the images. By fitting Gaussian curves to the profile lines, the laser beam diameter $d_{86}$ for each wavelength was estimated. The values for the vertical and horizontal profile and the mean values are printed in each image. Using the magnification $M_{\mathrm{bmc}}$, the mean laser beam diameter ${\bar{d}}_{86}$ was estimated to be 21.53 mm, 21.12 mm, 20.30 mm and 20.14 mm for the laser wavelength 488 nm, 515 nm, 561 nm and 640 nm, respectively. Since the beam profiles did not exhibit a distinctive ellipticity, we used these mean beam diameters for the data analysis of our stray light measurements (see Section 5).

### Appendix B.2. Characterization of the Camera

For the analyses of the images acquired during the experiments, it was essential to characterize the camera before the measurements. This comprised the verification of linearity and the estimation of its characteristics (see Table 2): saturation gray value $\mu_{y.\mathrm{sat}}$, absolute sensitivity threshold $\mu_{y.\min}$, overall system gain K and the quantum efficiency η. The characterization was conducted in accordance with the EMVA 1288 standard [15]. However, we performed only measurements necessary to estimate the aforementioned parameters and not the complete procedure for a valid camera characterization corresponding to the EMVA 1288 standard.

Figure A3 shows a schematic drawing of the experimental setup for the characterization. The illumination of the imaging sensor was provided by a light-emitting diode LED (Thorlabs M625F2) connected to an integrating sphere IS (Labsphere 3P-GPS-033-SL). In accordance with EMVA 1288 standard, the camera C was located at a distance to the exit port of the integrating sphere that equals eight times the exit port’s diameter. To prevent illumination of the imaging sensor from extraneous light, the beam path between the exit port of the IS and the camera was enclosed by a tube. Additionally, the lighting of the laboratory was switched off during measurements. A second exit port of the IS was used to connect a bifurcated fiber bundle (Thorlabs BFY400LS02). The two legs of the fiber bundle were connected to a reference photodiode PD (Ophir PD300R-UV) and a spectrometer SM (RGB Photonics Qwave).

Before the actual measurements were performed, the reference photodiode PD had to be calibrated by placing a power meter at the position of the camera’s imaging sensor and measuring the ratio of the signals when the LED was switched on. Using this ratio, we were able to calculate the irradiance at the position of the imaging sensor during the measurement by the readings of the reference photodiode. Furthermore, we could monitor whether the irradiance was kept constant during the measurement. Using the spectrometer SM, we measured a peak emission wavelength of 635 nm and a full width at half maximum (FWHM) spectral width of 20 nm for the LED.

For the characterization of the camera, we acquired a series of images for 56 different settings of the camera’s exposure time from 2800 µs (all pixel saturated) to the minimum of 60 µs in steps of 50 µs. For each setting, four images were acquired: two each with and without irradiation (dark frames). Using this image data, we calculated the mean gray value $\mu_{y}$ and the temporal variance of the gray values $\sigma_{y}^{2}$ for the illuminated images and the corresponding quantities $\mu_{y.\mathrm{dark}}$ and $\sigma_{y.\mathrm{dark}}^{2}$ for the dark frames. The specification to calculate these quantities is given by Equations (28) and (29) of the EMVA 1288 standard [15]. Furthermore, the mean number of photons $\mu_{p}$ per pixel was calculated using Equation (2) of the EMVA 1288 standard: $\mu_{p}=AEt_{\exp}/\left(hc/\lambda \right)$. Using these quantities, the necessary camera parameters could be derived.

Figure A4 shows a plot of the so-called photon transfer curve, where the photo-induced variance $\sigma_{y}^{2}-\sigma_{y.\mathrm{dark}}^{2}$ is plotted as blue data points versus the mean photo-induced gray value $\mu_{y}-\mu_{y.\mathrm{dark}}$. From this plot, the saturation gray value $\mu_{y.\mathrm{sat}}$ and the overall system gain K of the camera can be extracted. The saturation gray value is given as the mean gray value where the variance $\sigma_{y}^{2}-\sigma_{y.\mathrm{dark}}^{2}$ has its maximum (marked by a green data point in the plot); which is $\mu_{y.\mathrm{sat}}=3861\;\mathrm{DN}$ for our camera. The overall system gain is given by the slope of the curve. We estimated the overall system gain to be $K=0.399261\;\mathrm{DN}/e^{-}$ by a fit of a linear curve to the photon transfer curve (dashed line in the plot). The data range used for the fit was the data between the minimum value of $\mu_{y}-\mu_{y.\mathrm{dark}}$ and $0.7\cdot \mu_{y.\mathrm{sat}}$, marked by red data points in the plot.

In Figure A5, the sensitivity curve of the camera is shown. In this figure, the mean photo-induced gray value $\mu_{y}-\mu_{y.\mathrm{dark}}$ is plotted versus the mean number of photons $\mu_{p}$ arriving at a pixel. The slope of the linear part of this curve is the responsivity R of the camera. This value can also estimated by the fit of a linear curve to the data using the same data range as before and was estimated to be R=0.30595 DN/photon. The responsivity R and the overall system gain K are related by R=ηK. Using this relation the quantum efficiency at the wavelength of 635 nm was calculated to be $\eta \approx 0.77\;e^{-}/\mathrm{photon}$.

Using the number of photons $\mu_{p}$ corresponding to the saturation gray value $\mu_{y.\mathrm{sat}}$, we could calculate the saturation capacity $\mu_{p.\mathrm{sat}}\approx 12,488\;\mathrm{photons}$. The saturation capacity of our camera related to electrons is $\mu_{e.\mathrm{sat}}=\eta \cdot \mu_{p.\mathrm{sat}}\approx 9570\;e^{-}$.

From the sensitivity plot of Figure A5, we could also see that the linearity of the camera is quite good, which means that the camera was well suited for the kind of measurements presented in this publication.

## Appendix C

**Table A9:** Settings of the FRED software parameters for the stray light simulation.

**Light Source**		
Wavelength		550 nm
Beam shape		circular
Beam diameter	Fr1	32.0 mm
	Fr2	18.0 mm
	Fr3	20.1 mm
Input rays	Fr1/Fr3	cross-sectional: 31
		total: 749
	Fr2	cross-sectional: 35
		total: 973
Power		2 µW
Power distribution		Gaussian, $d_{86}=21.1\;\mathrm{mm}$
		
**Scatter Model (Lenses)**		
Model		Harvey Shack
Scatter parameters	Fr1/Fr2	s=−1.86; $b=0.36\;{\mathrm{sr}}^{-1}$; $b_{0}=6.92$; l=2.04 mrad (generic set as stated above)
	Fr3	s=−2.22; $b=1.09\;{\mathrm{sr}}^{-1}$; $b_{0}=16.14$; l=3.02 mrad (see Table 7, results for lens #8)
Scatter rays	Fr1	1,500,000
	Fr2	1,500,000
	Fr3	5,000,000
Importance sampling		11° to specular direction (semi-angle)
Direction type		uniform distribution
		
**Scatter Model (Housing, Aperture, Lens Edges)**		
Model		Black Lambertian
Reflectivity		4%
Importance sampling		default
		
**Sensor**		
Size		5.5 mm × 5.5 mm
Pixel		1000 × 1000
Pixel size		5.5 µm
Position		Best geometric focus
